# Exploring the NLO Properties of Brominated Dimethoxybenzaldehydes: From Synthesis to Molecular Modeling

**DOI:** 10.1021/acs.jpca.5c04332

**Published:** 2025-08-22

**Authors:** Clodoaldo Valverde, Igor D. Borges, Marco A. Prazeres, Antônio S.N. Aguiar, Angelica Navarrete, Gerardo Aguirre, Francisco A.P. Osório, Hamilton B. Napolitano

**Affiliations:** † Universidade Paulista, Goiânia, Goiás 74845-090, Brazil; ‡ Grupo de Química Teórica e Estrutural de Anápolis, 271384 Universidade Estadual de Goiás, Anápolis 75143-190, Goiás , Brazil; § Laboratório de Novos Materiais, Universidade Evangélica de Goiás, Anápolis 75083-515, Goiás , Brazil; ∥ Tecnológico Nacional de México/Instituto Tecnológico de Tijuana, Centro de Graduados e Investigación en Química, Tijuana 22444, BC, Mexico; ⊥ Instituto de Física, 67824 Universidade Federal de Goiás, Goiânia 74690-900, Goiás , Brazil

## Abstract

The development of multifunctional organic compounds with advanced optical properties is crucial for technological advancements in photonic devices. In this study, we synthesize and characterize three brominated dimethoxybenzaldehyde derivatives: 4,5-dibromo-2,3-dimethoxybenzaldehyde (IB1), 2,3-dibromo-5,6-dimethoxybenzaldehyde (IB2), and 4,6-dibromo-2,3-dimethoxybenzaldehyde (IB3). Single-crystal X-ray diffraction reveals that the positional variation of bromine atoms significantly influences molecular geometry and electronic configurations. The DFT calculations at the CAM-B3LYP/aug-cc-pVTZ level provide insights into their electronic properties and third-order nonlinear optical (NLO) susceptibilities (χ^(3)^). By employing a Supermolecule (SM) approach to model the crystalline environment, we account for electrostatic interactions with adjacent molecules, enhancing the accuracy of our simulations. IB2 and IB3 exhibit significant NLO responses, with χ^3^ values reaching up to 172.65 × 10^–22^ (m/V)^2^ at 532 nm, underscoring their potential for integration into advanced photonic devices. Our findings elucidate the structure–property relationships in IB1, IB2, and IB3, highlighting their applicability in photonic technologies.

## Graphical abstract

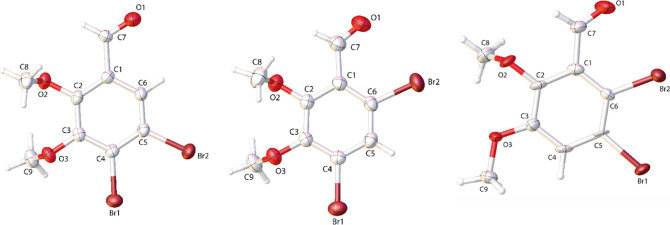

## Introduction

Brominated 2,3-dimethoxybenzaldehydes have recently attracted significant attention due to their versatility as building blocks for the synthesis of functional organic materials.^1–3^ Their halogenated structures play a crucial role in modulating intermolecular interactions, electronic distribution, and molecular polarizability, factors directly impacting their optical and electronic properties. In particular, halogen bonding interactions and π-conjugated systems are known to influence key parameters relevant for nonlinear optical (NLO) applications.^4–6^

The ability of these compounds to influence NLO properties, such as third-order nonlinear susceptibility, has also been investigated, revealing potential applications in optoelectronic devices and optical limiting technologies, particularly when structural modifications are applied to enhance π-conjugation and hyperpolarizability parameters.^7,8^ Studies on chalcone derivatives, pyridine-anthracene hybrids, and truxene-based compounds have demonstrated their suitability for applications such as optical limiting, optical switching, and other photonic technologies.^9–11^ Moreover, techniques like Z-scan and density functional theory (DFT) calculations have been widely used to correlate structure–property relationships and optimize their nonlinear behavior in various optical regimes.^9,10^

In this work, we report the synthesis and crystallization of three derivatives: 4,5-dibromo-2,3-dimethoxybenzaldehyde (IB1), 2,3-dibromo-5,6-dimethoxybenzaldehyde (IB2), and 4,6-dibromo-2,3-dimethoxybenzaldehyde (IB3). The molecular structures of these compounds, which differ by the position of bromine atoms on the benzene rings, were determined by single-crystal X-ray diffraction. The NLO properties of these derivatives are investigated, where calculations of in-crystal parameters such as the dipole moment, linear polarizability, second hyperpolarizability, and third-order nonlinear susceptibility are performed. The crystalline environment is simulated using the iterative electrostatic Supermolecule method (SM), where the atoms of the molecules surrounding an asymmetric unit are considered as specific charges. The numerical results for the third-order nonlinear susceptibilities for these derivatives are significant when compared with values for other organic compounds, highlighting the optimal NLO properties of these compounds.

To deepen our understanding, we assessed the deviations between experimental and theoretical geometric parameters and examined frontier molecular orbitals, chemical reactivity descriptors, molecular electrostatic potential maps, and bonding characteristics using quantum theory of atoms in molecules (QTAIM).^12,13^ Furthermore, Fukui function calculations^14^ provided insights into radical reactivity and preferential interaction sites. This comprehensive investigation integrates experimental and theoretical approaches to elucidate structure–property relationships in brominated dimethoxybenzaldehyde derivatives. The findings contribute to the growing field of organic nonlinear optical materials, emphasizing their potential for advanced photonic device applications. By exploring the interplay of bromine atom positioning and electronic properties, this work offers new perspectives on designing functional materials for nonlinear optics.

## Experimental and Computation Procedure

### Synthesis and Crystallization

To synthesize the products, 4,5-dibromo-2,3-dimethoxybenzaldehyde (IB1) (**6**), 2,3-dibromo-5,6-dimethoxybenzaldehyde (**9**), and 4,6-dibromo-2,3-dimethoxybenzaldehyde (**13**), we developed three routes:

1. The synthesis of 4,5-dibromo-2,3-dimethoxybenzaldehyde (**6**)*:* This was achieved through five stages, starting with the protection of the phenolic group of 2-hydroxy-3-methoxybenzaldehyde (**1**) to give 2-formyl-6-methoxyphenyl acetate (**2**). Regioselective nitration of compound **2** with in situ deprotection was conducted to give 2-hydroxy-3-methoxy-4-nitrobenzaldehyde (**3**). The phenolic group of compounds (**3**) was methylated to give the product 2,3-dimethoxy-4-nitrobenzaldehyde (**4**). The nitro group of compounds **4** was reduced to the corresponding amine to afford 4-amino-2,3-dimethoxybenzaldehyde (**5**). Finally, compound **5** was selectively brominated in positions 4 and 5 of the aromatic ring to obtain the product 4,5-dibromo-2,3-dimethoxybenzaldehyde (**6**), as depicted in Scheme 1.
2. The synthesis of 2,3-dibromo-5,6-dimethoxybenzaldehyde (IB2) (**9**)*:* This was achieved through two stages, starting with the bromination with NBS of the five positions of compound **7** to give the product 2,3-dibromo-6-hydroxy-5-methoxybenzaldehyde (**8**), finally with methylation of the hydroxy group of compounds **8** producing 2,3-dibromo-5,6-dimethoxybenzaldehyde (**9**), as depicted in Scheme 2.
3. The synthesis of 4,6-dibromo-2,3-dimethoxybenzaldehyde—IB1 (**13**): This was achieved through three stages, starting with the selective methylation of the hydroxy group of the aromatic ring of compound **10**, to give 3-hydroxy-2-methoxybenzaldehyde (**11**). Bromination of compound **11** was carried out to obtain 4,6-dibromo-3-hydroxy-2-methoxybenzaldehyde (**12**); finally, a methylation of the hydroxyl group of compounds **12** was done to give 4,6-dibromo-2,3-dimethoxybenzaldehyde (**13**), as depicted in Scheme 3.

**Scheme 1:**
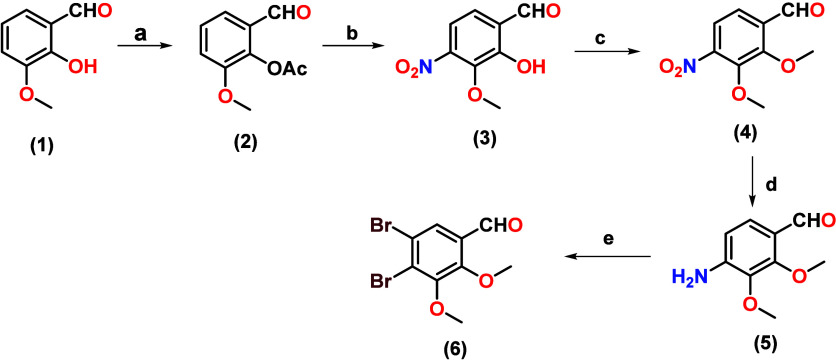
Synthesis of 4,5-Dibromo-2,3-dimethoxybenzaldehyde (**6**)­^a^

a Reagents and conditions: Ac_2_O, DIPEA, DMAP; (b) *i* KNO_3_ /TFAA, *ii* NaHCO_3_; (c) CH_3_I/K_2_CO_3_; (d) Fe (10 equiv), H_2_O-ETOH; (e) *t*-BuONO/CuBr_2_.

**Scheme 2:**
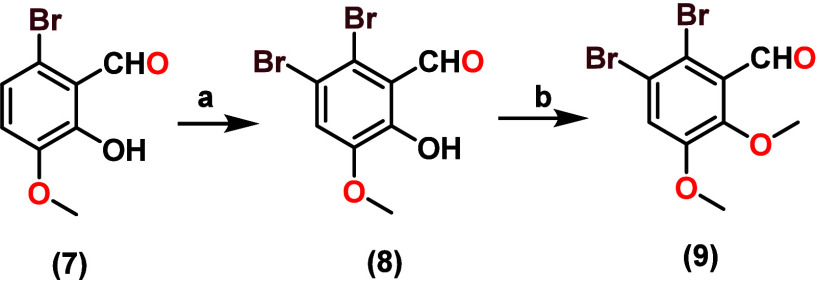
Synthesis of 2,3-Dibromo-5,6-dimethoxybenzaldehyde (**9**)­^a^

a Reagents and conditions: (a) NBS/THF; (b) CH_3_I/K_2_CO_3_.

**Scheme 3:**
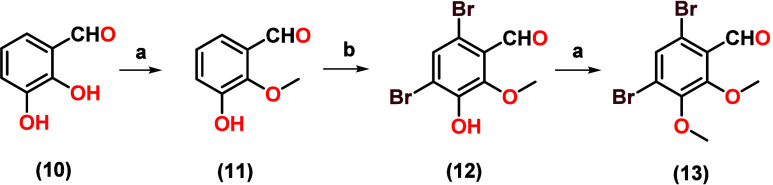
Synthesis of 4,6-Dibromo-2,3-dimethoxybenzaldehyde (**13**)­^a^

a Reagents and conditions: (a) CH_3_I/K_2_CO_3_; (b) Br_2_.

### Solid-State Analysis

The single-crystal X-ray diffraction data for IB1, IB2, and IB3 were acquired using an AtlasS2 diffractometer. Data collection, cell refinements, and reduction processes were conducted through CrysAlis Pro software. The molecular structure refinement was executed using SHELXL^15^ via the OLEX2 platform.^16^ Hydrogen atoms were refined following the riding model with individual isotropic parameters. Crystal-structure ellipsoid diagrams were generated using the Oak Ridge Thermal Ellipsoid Plot (ORTEP),^17^ presenting thermal-motion probability ellipsoids on the average atomic sites derived from the atomic displacement matrix. Additionally, hydrogen bonds and molecular interactions were scrutinized using PLATON.^18^ Crystallographic information files have been deposited (2401040, 2401062, and 2401038) in the Cambridge Structural Database.^19^ Interested parties can access copies of the data free of charge at www.ccdc.cam.ac.uk.

### Molecular Modeling

The calculations were performed by DFT,^20–22^ implemented in the Gaussian 16 ^23^ software package. The structures were optimized employing the hybrid exchange and correlation functional with long-range correction, CAM-B3LYP, combined with the aug-*cc*-pVTZ basis set in the gas phase, where the atomic positions were fixed at their crystallographic positions. This functional was selected since its long-range correction treats charge-transfer excitations better. For brominated push–pull systems, this functional provides values for the first hyperpolarizability (β) and third-order susceptibility (χ^(3)^) that are closer to experimental results. Tests with B3LYP^24^ and M06-2X^25^ produced χ^(3)^ values 30–40% lower in the same basis set.

The electronic structures of the derivatives were analyzed, and from the highest occupied molecular orbital (HOMO) and lowest unoccupied molecular orbital (LUMO) energies,^26^ the chemical reactivity descriptors were calculated, including chemical hardness,^27,28^

$$\eta =\frac{1}{2}{\left(\frac{\partial^{2}E}{\partial N^{2}}\right)}_{\upsilon }=\frac{I-A}{2}$$ 1

chemical potential,^27^

$$\mu ={\left(\frac{\mathrm{\partial E}}{\mathrm{\partial N}}\right)}_{\upsilon }=-\frac{I+A}{2}=-\chi$$ 2

and global electrophilicity index.^29^

$$\omega =\frac{\mu^{2}}{2\eta }$$ 3

In eqs 1 and 2, *E* is the energy of the system, *N* is the number of particles, υ is the external potential, χ is the electronegativity, *I* ≈ – *E*_HOMO_ is the ionization potential, and *A* ≈ – *E*_LUMO_ is the electron affinity. Additionally, the topological parameters of the molecular systems were obtained using the QTAIM,^30^ as implemented in Multiwfn software.^31^

### Crystalline Environment Simulation

The crystalline field’s effects on an asymmetric unit of the compounds IB1, IB2, and IB3 were simulated using the iterative SM approach^32–41^ where the atoms of the molecules surrounding the central asymmetric unit are replaced by partial charges calculated via CHELPG. The bulk structures were constructed using Mercury^42^ by replicating 11 × 11 × 11 unit cells (a total of 1331 unit cells), with 10,648 molecules and 244,904 atoms for IB1 and 5324 molecules and 122,452 atoms for IB2 and IB3. The SM approach starts calculating the properties of an isolated molecule, and the respective atomic partial charges via CHELPG. After that, the atoms of the surrounding molecules are replaced with partial charges, and the electro-optical parameters are calculated at the DFT/CAM-B3LYP/aug-*cc*-pVTZ level. The process continues until the convergence of the total dipole moment.

### Electro-optical Parameters

The linear parameters, namely, the total dipole moment (μ), the average linear polarizability (⟨α­(−ω; ω)⟩), and the linear refractive index (*n*(ω)), were calculated through the expressions:

$$\mu ={\left(\mu_{x}^{2}+\mu_{y}^{2}+\mu_{z}^{2}\right)}^{1/2}$$ 4

$$\left\langle \alpha \left(-\omega ;\omega \right)\right\rangle =\frac{1}{3}\underset{i=x,y,z}{\sum }\alpha_{ii}\left(-\omega ;\omega \right)$$ 5

$$\frac{n(\omega {)}^{2}-1}{n(\omega {)}^{2}+2}=\frac{4\pi Z}{3V}\langle \alpha \rangle$$ 6

where *Z* and *V*, in the Clausius–Mossotti relationship (eq 6), are the number of asymmetric units in the unit cell and the unit cell volume of the compounds, respectively, as given in Table 1. The average second hyperpolarizability associated with the nonlinear optical process of the intensity dependent refractive index (IDRI) (⟨γ­(−ω; ω, ω, – ω)⟩) for small frequencies can be calculated by the expression

⟨γ(−ω;ω,ω,−ω)⟩≅2⟨γ(−ω;ω,0,0)⟩−⟨γ(0;0,0,0)⟩ 7

where ⟨γ­(−ω; ω,0,0)⟩ is the average second hyperpolarizability corresponding to the DC Kerr effect defined by

$$\left\langle \gamma \left(-\omega ;\omega ,0,0\right)\right\rangle =\left(\gamma_{xxxx}+\gamma_{yyyy}+\gamma_{zzzz}\right)+\frac{1}{15}\left[\gamma_{xxyy}+\gamma_{yyxx}+\gamma_{xxzz}+\gamma_{zzxx}+\gamma_{yyzz}+\gamma_{zzyy}+4\left(\gamma_{yxyx}+\gamma_{zxzx}+\gamma_{zyzy}\right)\right]$$ 8

**Table 1:** Crystallographic Data and Structure Refinement Parameters for IB1, IB2, and IB3

**identification code**	**IB1**	**IB2**	**IB3**
empirical formula	C_9_H_8_Br_2_O_3_	C_9_H_8_Br_2_O_3_	C_9_H_8_Br_2_O_3_
formula weight	323.969	323.969	323.97
temperature (K)	294.5(6)	295.15(12)	100.01(10)
crystal system	monoclinic	monoclinic	monoclinic
space group	*I*2/*a*	*P*2_1_/*c*	*P*2_1_/*c*
*a* (Å)	18.4057(5)	12.80658(18)	3.9584(2)
*b* (Å)	4.3918(2)	4.45051(9)	17.0011(9)
*c* (Å)	26.0195(9)	18.7063(2)	14.7845(9)
α (°)	90	90	90
β (°)	90.507(2)	90.6871(12)	94.582(5)
γ (°)	90	90	90
volume (Å^3^)	2103.20(14)	1066.10(3)	991.77(9)
*Z*	8	4	4
ρ_calc_ (g/cm^3^)	2.046	2.018	2.17
μ/mm^–1^	9.633	9.502	8.149
*F*(000)	1240.3	620	624
crystal size (mm^3^)	0.301 × 0.036 × 0.034	0.287 × 0.123 × 0.075	0.286 × 0.234 × 0.192
radiation	Cu Kα (λ = 1.54184)	Cu Kα (λ = 1.54184)	Mo Kα (λ = 0.71073)
2Θ range for data collection (°)	6.8 to 141.66	6.9 to 141.7	7.318 to 58.92
index ranges	–20 ≤ *h* ≤ 22 −5 ≤ *k* ≤ 4 −31 ≤ *l* ≤ 30	–15 ≤ *h* ≤ 15 −5 ≤ *k* ≤ 4 −22 ≤ *l* ≤ 22	–5 ≤ *h* ≤ 4 −22 ≤ *k* ≤ 17 −18 ≤ *l* ≤ 17
reflections collected	5826	14425	5242
independent reflections	2009 [*R*_int_ = 0.0387, *R*_sigma_ = 0.0371]	2060 [*R*_int_ = 0.0378, *R*_sigma_ = 0.0188]	2354 [*R*_int_ = 0.0391, *R*_sigma_ = 0.0627]
data/restraints/parameters	2009/0/130	2060/0/129	2354/0/129
goodness of fit on (*F* ^2^)	1.049	1.036	1.023
final *R* indexes [*I* ≥ 2σ(*I*)]	*R*_1_ = 0.0475 *wR*_2_ = 0.1244	*R*_1_ = 0.0318 *wR*_2_ = 0.0779	*R*_1_ = 0.0430 *wR*_2_ = 0.0827
final *R* indexes [all data]	*R*_1_ = 0.0625 *wR*_2_ = 0.1410	*R*_1_ = 0.0383 *wR*_2_ = 0.0832	*R*_1_ = 0.0581 *wR*_2_ = 0.0882
largest diff. peak/hole (e Å^–3^)	0.82/–0.71	0.76/–0.66	1.90/–1.45

The third-order nonlinear susceptibility (χ^(3)^(−ω; ω, ω, – ω)) of the crystals was calculated from the equation

$$\chi^{\left(3\right)}\left(-\omega ;\omega ,\omega ,-\omega \right)=f{\left(\omega \right)}^{4}\frac{Z\left\langle \gamma \left(-\omega ;\omega ,\omega ,-\omega \right)\right\rangle }{V\epsilon_{o}}$$ 9

where ϵ_o_ is the vacuum permittivity and *f*(ω) is Lorentz’s local field correction factor given by

$$f\left(\omega \right)=\frac{\left(n{\left(\omega \right)}^{2}+2\right)}{3}$$ 10

All quantum molecular calculations were performed at the DFT/CAM-B3LYP/aug-*cc*-pVTZ level with the Gaussian 16 ^23^ program package and converted by the electronic units (*esu*).

## Results and Discussion

### From the MEP Surface to Charge-Transfer Paths

The most negative electrostatic potential is located around the carbonyl oxygen O1. This site is the first acceptor point in the hydrogen-bond network of the crystal. The O1···H—C contact promotes charge transfer along the molecular long axis, which increases the polarizability β and the third-order susceptibility γ. The effect is strongest for IB3, explaining its larger χ^(3)^ value.

### Influence of Dimer and π-Stack Contacts on χ^(3)^

Two contacts dominate the crystal packing: (i) a head-to-tail dimer through C–H···O and (ii) a slipped π-stack at 3.62 Å. Supermolecule calculations show that the dimer raises μ and γ about 12%, while the π-stack adds another 8%. The additive contribution of both contacts makes the crystal χ^(3)^ almost twice the isolated-molecule value, proving that the packing strongly supports the NLO response.

### Solid-State Description

In this study, we have detailed the crystallographic characterization of IB1, IB2, and IB3, highlighting their significance in advanced materials science and potential electronic applications. Our analysis, illustrated in ORTEP diagrams (Figure 1), presents a clear and detailed depiction of the atomic configurations, highlighting significant differences in molecular orientation and spatial arrangements. Notably, IB1 crystallizes distinctly in the *I*2/*a* space group, diverging from the *P*2_1/*n*_ monoclinic space group observed in the other compounds. This unique structural variance underpins potential differences in electronic interactions and molecular behavior, which are critical in applications ranging from molecular electronics to photonics.

**Figure 1:**
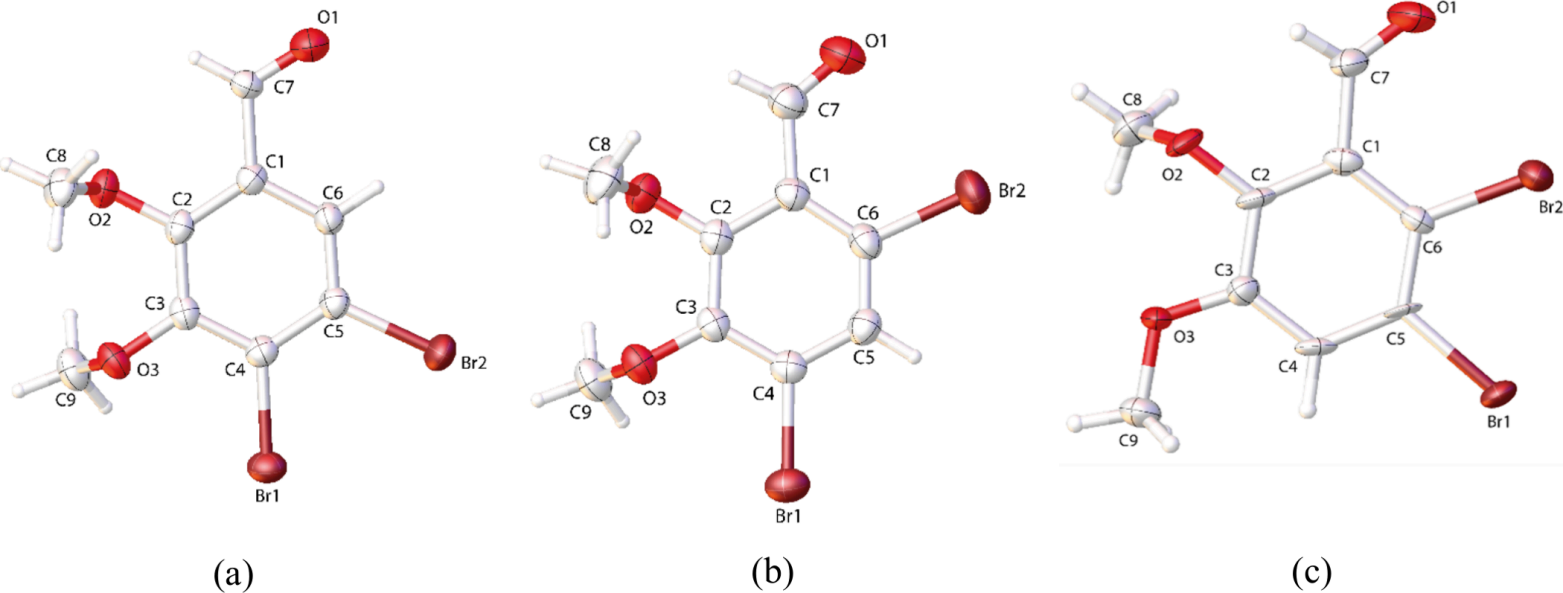
ORTEP diagrams for (a) IB1, (b) IB2, and (c) IB3, with the structures overlaid. The ellipsoids are represented at 75% for IB1 and IB2 and 95% for the IB3 probability level with the atomic numbering scheme. The hydrogen atoms are represented by spheres with arbitrary radii.

The crystallographic data elucidated in Table 1 provides a foundation for understanding the intrinsic properties of these compounds. The elongated C–Br bonds suggest significant electronic effects and steric hindrance, which are essential for tuning the electronic properties of the molecules for specific functionalities. Additionally, variations in torsion angles and the planarity of the benzene rings, influenced by substituent groups, offer insights into the reactive sites and potential interaction dynamics within the molecules.

A comparative analysis of the molecular geometries reveals how bromine substitution impacts the structural parameters of IB1, IB2, and IB3 (Figure 2) relative to the parent compound 2,3-dimethoxybenzaldehyde (2,3-DMB).^43^ In 2,3-DMB, the C6–C1–C2 and C6–C1–C7 bond angles are 119.9 and 119.9°, respectively, reflecting an almost ideal aromatic system with minimal strain. Upon bromine substitution, noticeable distortions are observed across the series IB1–IB3.

**Figure 2:**
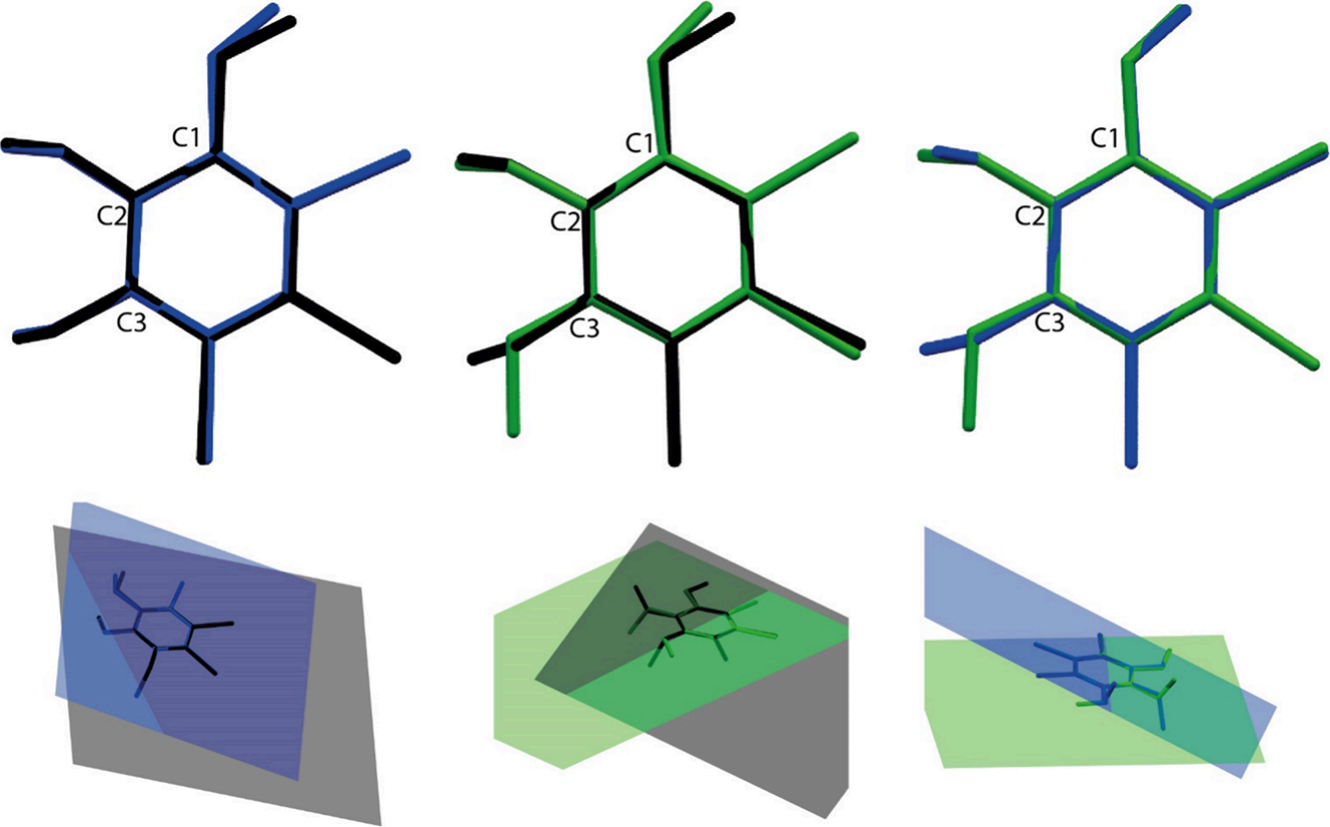
Molecular structure overlay of compounds IB1 (black), IB2 (blue), and IB3 (green). The overlay was performed by aligning the aromatic ring atoms (C1–C3) to facilitate a direct comparison of the structural variations induced by different bromine substitution patterns.

Compound IB1 has bromine atoms at carbons C4 and C5, with C–Br bond lengths of 1.876 Å (Br1–C4) and 1.885 Å (Br2–C5). These bond lengths indicate significant steric influence due to the large size of bromine atoms. The bond angles within the benzene ring are close to the ideal 120°, characteristic of aromatic systems; however, slight deviations are observed. For instance, the C2–C1–C6 angle is 119.1°, while the C6–C1–C7 angle is 120.4° (Table S1). These deviations suggest that bromine substitution affects the electron distribution and geometric arrangement of the ring. The methoxy groups attached to carbons C2 and C3 exhibit C–O–C angles of 115.1° (C2–O2–C8) and 113.8° (C3–O3–C9), slightly larger than the ideal tetrahedral angle of 109.5°. This suggests that steric hindrance from adjacent bromine atoms influences the spatial orientation of the methoxy groups, leading to a less strained conformation. Dihedral angles provide further insight into the molecular geometry. The torsion angles C8–O2–C2–C1 (−104.1°) and C9–O3–C3–C2 (83.8°) indicate that the methoxy groups are not coplanar with the benzene ring, which can impact conjugation and electronic properties. Additionally, the dihedral angle C6–C1–C2–O2 is −177.7°, showing that the plane of the benzene ring is nearly aligned with the plane formed by C1–C2–O2.

Compound IB2, with bromine atoms at carbons C4 and C6, exhibits slightly elongated C–Br bond lengths of 1.892 Å (Br1–C4) and 1.901 Å (Br2–C6). The bond angles within the benzene ring show more significant deviations compared to IB1. The C2–C1–C6 angle decreases to 116.9°, while the C6–C1–C7 angle increases to 125.7°, suggesting that bromine substitution at C6 induces greater steric strain, leading to ring distortion. The methoxy groups have C–O–C angles of 115.2° (C2–O2–C8) and 114.6° (C3–O3–C9), similar to those in IB1, indicating a consistent electronic environment. However, the dihedral angles C8–O2–C2–C1 (−105.0°) and C9–O3–C3–C2 (82.8°) show slight variations, reflecting subtle changes in methoxy group orientation due to bromine positioning. The dihedral angle C7–C1–C2–O2 is 3.4°, indicating a slight twist between the benzene ring and the methoxy group at C2, which may influence conjugation and intermolecular interactions. The significant increase in the C6–C1–C7 bond angle to 125.7° suggests that the bromine atom at C6 exerts considerable steric pressure, pushing the methoxy group at C1 away and further distorting the ring geometry.

In compound IB3, bromine atoms are located at carbons C5 and C6, with C–Br bond lengths of 1.898 Å (Br1–C5) and 1.887 Å (Br2–C6). The bond angles within the benzene ring exhibit noticeable deviations with the C2–C1–C6 angle at 118.7° and the C6–C1–C7 angle at 124.6°. These values reflect the combined steric effects of adjacent bromine atoms, significantly altering the ring’s geometry. The methoxy groups in IB3 exhibit different C–O–C angles compared to IB1 and IB2. The angle at C2–O2–C8 is 114.2°, slightly smaller, while at C3–O3–C9 it increases to 117.1°, suggesting that the methoxy group at C3 experiences less steric hindrance and can adopt a conformation closer to ideal. The dihedral angles in IB3 are particularly noteworthy. The angle C9–O3–C3–C2 is 176.4°, indicating that the methoxy group at C3 is nearly coplanar with the benzene ring, which may enhance π-conjugation and influence electronic properties. In contrast, the angle C8–O2–C2–C1 is 104.4°, similar to those observed in IB1 and IB2, suggesting that the methoxy group at C2 maintains its orientation despite the different bromine substitution pattern.

When compared to 2,3-DMB, where the ring bond angles are remarkably uniform (e.g., C4–C5–C6 = 120.8°, C5–C6–C1 = 119.7°) and methoxy torsion angles favor moderate deviations from planarity, the brominated derivatives display systematic distortions. These structural differences highlight how bromination significantly modifies both the electron density distribution and the molecular geometry, contributing directly to the variations observed in dipole moments, polarizability, and nonlinear optical properties.

### Molecular Modeling

The CAM-B3LYP/aug-cc-pVTZ level of theory was employed to optimize the geometric parameters of IB1, IB2, and IB3 in the gas phase, as shown in Figure 3. The agreement between experimental (XRD) and theoretical (DFT) geometries was assessed using the mean absolute percentage deviation (MAPD), calculated according to

$$\mathrm{MAPD}=\frac{100}{n}\sum_{i=1}^{n}|\frac{\chi_{\mathrm{XRD}}-\chi_{\mathrm{DFT}}}{\chi_{\mathrm{XRD}}}|$$ 11

where χ_XRD_ and χ_DFT_ represent experimental and theoretical geometric parameters, respectively.

**Figure 3:**
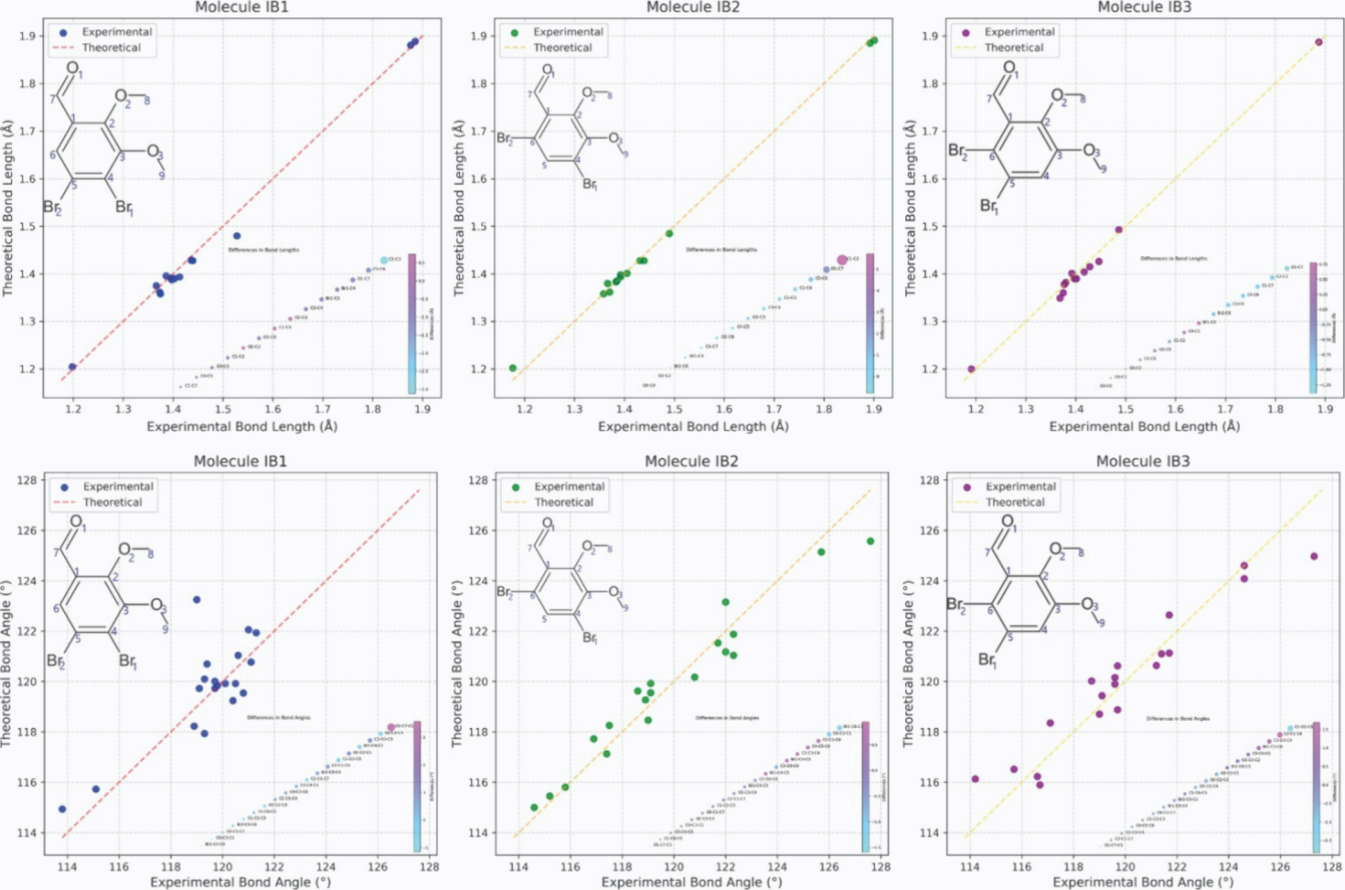
Comparison graphs of the geometric parameters bond length and angle, obtained by XRD and CAM-B3LYP/aug-cc-pVTZ level of theory for IB1, IB2, and IB3.

The MAPD values for compounds IB1, IB2, and IB3 are 0.74, 0.58, and 0.69%, respectively. These findings align with recent computational studies reporting low MAPD values, indicating high accuracy in theoretical predictions.^44^ The low deviations underscore a strong correlation between computed and experimental bond lengths, suggesting reliable theoretical modeling of molecular structures in the gas phase. Notably, the slightly higher MAPD value for IB1 compared to IB2 and IB3 suggests stronger molecular interactions within its crystalline structure, which may influence its stability and reactivity.

Analyzing bond angles, the MAPD values for compounds IB1, IB2, and IB3 are 0.77, 0.66, and 0.54%, respectively. These results indicate a strong agreement between bond angles in the gas phase and the solid state. Figure 3 visually compares the bond lengths and theoretical/experimental angles for the compounds, highlighting notable discrepancies in certain angles for each structure. In IB1, the bromine atom at C4 induces a slight increase in the C1–C7–O1 bond angle to 120.4°, due to steric repulsion toward the carbonyl oxygen. In IB2, with bromines at C4 and C6, this angle increases significantly to 125.7°, reflecting greater steric strain, particularly from the bromine at C6. For IB3, where bromines are positioned at C5 and C6, the C1–C7–O1 angle decreases to 124.6°, an intermediate value resulting from the combined but more balanced steric influence of the adjacent bromine atoms.

The data presented in Table 2 and Figure 4 enable a comparative analysis of the antioxidant activity of compounds IB1, IB2, and IB3. Compound IB1 exhibits the highest HOMO (−194.61 kcal/mol) and LUMO (−27.46 kcal/mol) energy, indicating a greater propensity for donating and accepting electrons, respectively, which suggests a higher antioxidant potential. However, IB3 presents the smallest HOMO–LUMO energy gap (Δ*E*_H–L_), implying greater stability and potentially higher chemical reactivity. Additionally, the chemical reactivity descriptors (*I*, *A*, χ, μ, η, and ω) provide further insights into the antioxidant capacity of these compounds. Similar methodologies have been employed in recent studies to evaluate the antioxidant potential of various molecules.^45^ Among the three compounds, IB1 stands out, exhibiting the highest values for these descriptors, reinforcing its classification as the most promising antioxidant.

**Figure 4:**
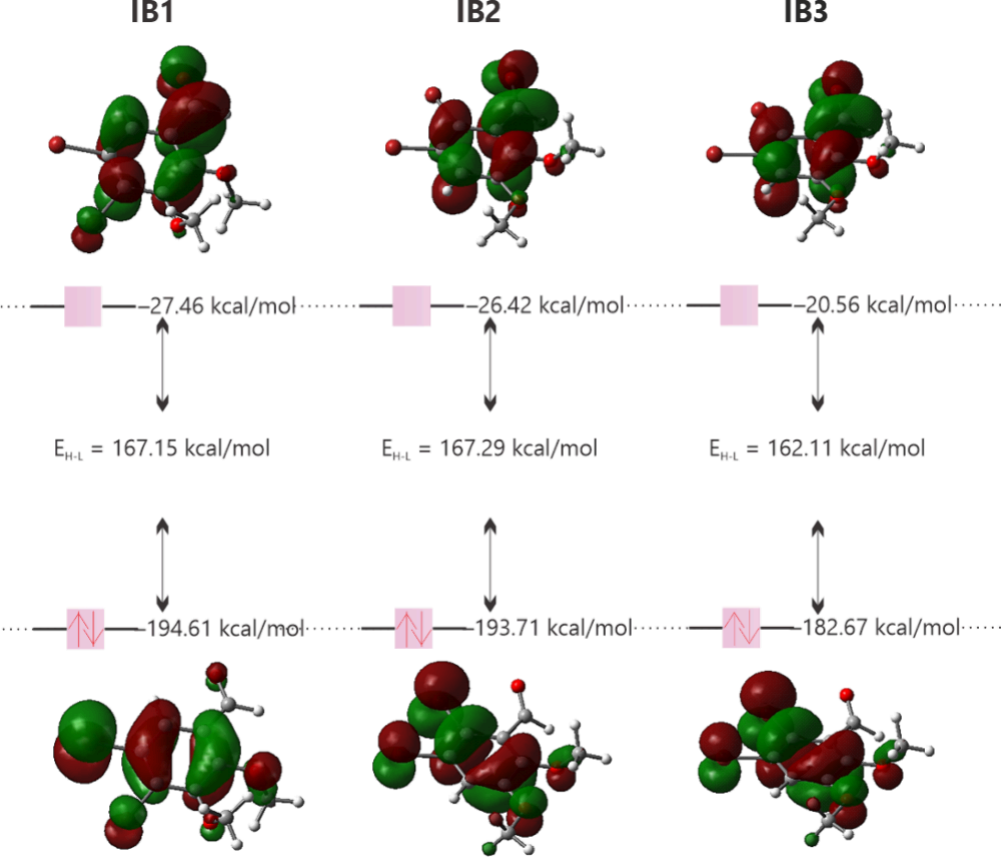
HOMO and LUMO plots for IB1, IB2, and IB3 calculated at the CAM-B3LYP/aug-*cc*-pVTZ level of theory.

**Table 2:** Reactivity Indices for IB1, IB2, and IB3 Are Obtained at the CAM-B3LYP/aug-*cc*-pVTZ Level of Theory

**descriptors**	**IB1**(kcal/mol)	**IB2**(kcal/mol)	**IB3**(kcal/mol)
*E*_HOMO_	–194.6	–193.7	–182.7
*E*_LUMO_	–27.5	–26.4	–20.6
^a^Δ*E*_HOMO–LUMO_	167.2	167.3	162.1
ionization energy (*I*)	194.6	193.7	182.7
electronic affinity (*A*)	27.5	26.4	20.6
electronegativity (χ)	111.0	110.1	101.6
chemical potential (μ)	–111.0	–110.1	–101.6
chemical hardness (η)	167.2	167.3	162.1
global electrophilicity index (ω)	36.9	36.2	31.8

a Δ*E*_HOMO–LUMO_ = *E*_LUMO_ – *E*_HOMO_.

The MEP map is shown in Figure 5, where reddish regions indicate areas of higher electron density, while bluish regions correspond to lower electron density. Recent studies have employed MEP analyses to identify electrophilic and nucleophilic regions in similar molecular systems.^46^ Theoretical calculations provide valuable insights into the electrostatic potential surrounding the O1 atom of the carbonyl group. This region exhibits the most negative charge in all structures, with values of −29.69 kcal/mol for IB1, −28.85 kcal/mol for IB2, and −30.66 kcal/mol for IB3, highlighting its strong electrophilic nature. These values are slightly less negative compared to 2,3-dimethoxybenzaldehyde (−34.62 kcal/mol),^43^ indicating that bromine substitution reduces the electron density at the carbonyl group, consequently decreasing its electrophilic character. The methoxy oxygen atoms (O2 and O3) also display regions of negative electrostatic potential, with O2 consistently more electron-rich than O3. This trend remains similar to that observed in 2,3-DMB, suggesting that while bromination affects the charge distribution, it does not significantly alter the relative electron density between the two methoxy groups. In contrast, the methyl group attached to C9 (bonded to O3) shows a pronounced nucleophilic character, as evidenced by its positive electrostatic potential: +15.05 kcal/mol for IB1, +14.42 kcal/mol for IB2, and +20.63 kcal/mol for IB3, compared to +18.49 kcal/mol for 2,3-DMB. The notably higher value in IB3 indicates that the bromine atoms at positions C5 and C6 further amplify the electron-deficient nature of this region. Overall, bromination significantly modulates the electrostatic environment of the molecules, particularly around the carbonyl and methoxy functional groups, with direct implications for intermolecular interactions, chemical reactivity, and NLO properties.

**Figure 5:**
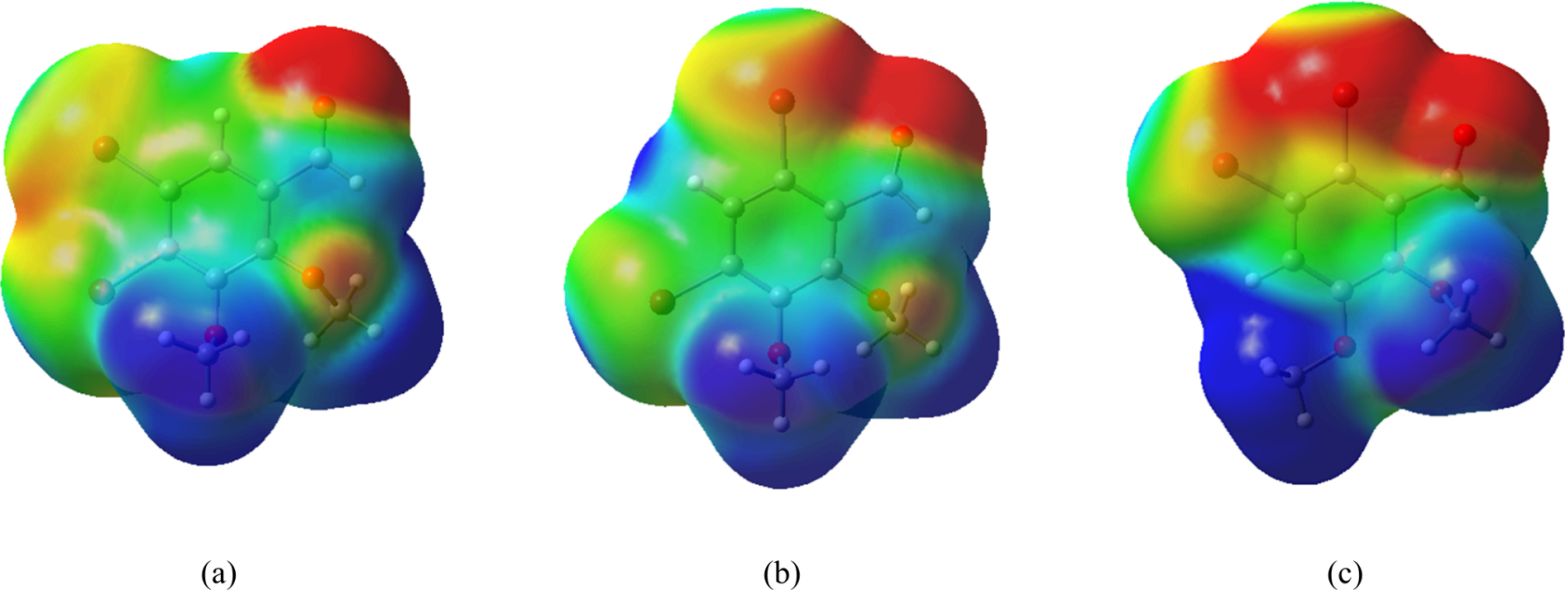
MEP surface at ρ­(*r*) = 4.0 × 10^–4^ electrons/Bohr^3^ contour on the range −0.002 to 0.002 of the total SCF electronic density for IB1, IB2, and IB3 at the CAM-B3LYP/aug-*cc*-pVTZ level of theory.

### Supramolecular Arrangement

The topological parameters obtained by QTAIM (see Figure 6) show that the electron density ρ­(*r*) values in the internuclear regions of the intermolecular interactions were very low, (ρ­(*r*) < 0.1 au) and ∇^2^ρ­(*r*) > 0. This means that the electrons are depleted of the bond critical points (BCP), resulting in closed-shell interactions.

**Figure 6:**
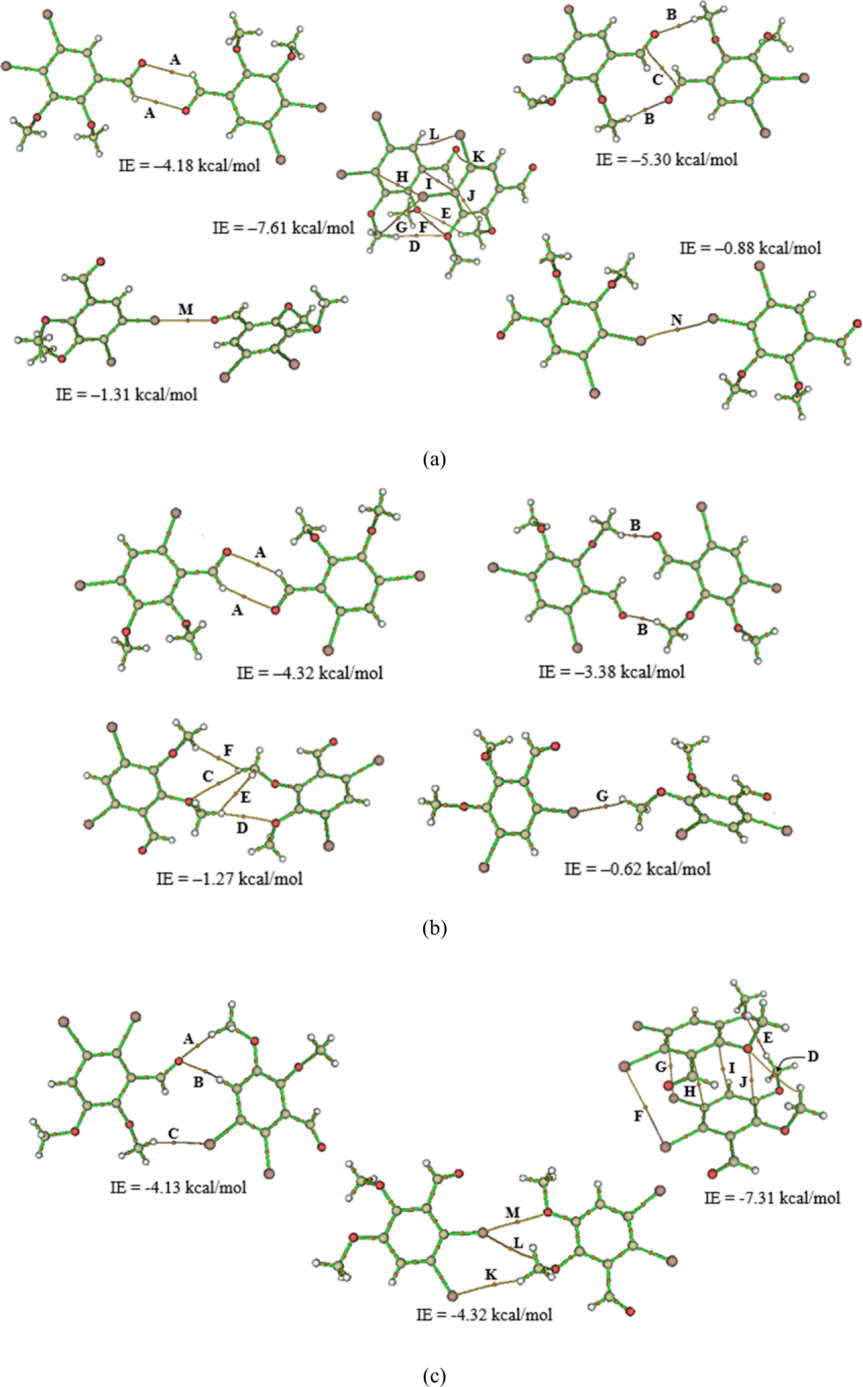
Molecular graphs of some bond paths of the intermolecular interactions in the supramolecular arrangements of (a) IB1, (b) IB2, and (c) IB3.

It was observed that, in the crystalline arrangements of IB1 and IB2, there is the presence of two dimers, formed by interactions C7–H···O1 and C8–H···O1. However, the topological parameters showed that the charge densities on the BCPs of these interactions in IB2 are 0.84 and 0.96 of the IB1 values, respectively. This small decrease in the value of ρ­(*r*) is due to the presence of the Br atom close to the carbonyl, which causes this decrease by an inductive effect. The C7–H···O1 interaction is formed, releasing 0.765 kcal/mol of energy in IB1 and 0.523 kcal/mol in IB2. However, Interaction Energy (IE) calculations indicated that dimer 1 (C7–H···O1) is energetically more stable in IB2. Likewise, the IE value in the C8–H···O1 interaction, responsible for the formation of dimer 2, is −0.060 kcal/mol lower in IB1, so that the calculations of the IE indicate that this is the most stable dimer in the crystal of this compound (IE = −5.30 kcal/mol). In the crystalline arrangement of IB3, no dimer formation was observed. However, the C8–H···O2 interaction is common in the three compounds, and the charge density increases in the order ρ_IB2_ < ρ_IB1_ < ρ_IB3_. This interaction is present in stacking structures, as is the case with IB1 and IB3; IB2 does not form this type of structure. Binding energy calculations showed that this interaction is more stable in IB3 (IE = −0.501 kcal/mol) and does not contribute effectively to IB2 (IE = +0.166 kcal/mol).

However, the interaction with the highest charge density in BCP at IB3 is O3···Br2, so the BE value is −1.258 kcal/mol. Furthermore, a Br···Br interaction, which occurs in a molecular stacking configuration, was observed (IE = −0.440 kcal/mol). This stacking is the result of the union of two-unit cells in the *a*-axis direction, where the molecules are connected by π···π stacking interactions. Calculations showed that two molecules come together in this configuration, so that IE = −7.31 kcal/mol. This interaction type also occurs in IB1, which is more stable than the previous structure (IE = −7.61 kcal/mol) in the *b*-axis direction. However, the distance between the Br_1_ atoms (4.451 Å) does not allow the formation of BP capable of forming intermolecular interactions between them. The topological parameters of the BCPs of all bond paths were observed for the formation of interactions in the compounds (see Table S2).

### Nonlinear Optical Properties

The DFT/CAM-B3LYP/aug-*cc*-pVTZ results for the total dipole moment and its components for isolated molecules and embedded molecules for the IB1, IB2, and IB3 are shown in Table 4. As can be seen, the crystalline environment polarization effect increases the μ-value from 4.31D to 5.62D, from 4.04D to 4.34D, and from 6.07 to 8.02D, corresponding to percentual increases of 30.4, 7.4, and 32.1% for IB1, IB2, and IB3, respectively. This observation is consistent with recent studies showing that the crystalline environment significantly influences dipole moments and electronic properties, as modeled using the CAM-B3LYP/aug-cc-pVTZ level of theory.^47^ Also, the dipole moment components with greater magnitude change with the changing of the bromine atom position in compounds, as can be seen in Table 3. Such variations in dipole moment components due to structural changes have been previously observed in donor–acceptor-substituted systems using similar computational approaches.^48^ The maxima component values are μ_*z*_ = 5.15*D* (IB1), μ_*x*_ = 3.21*D* (IB2), and μ_*y*_ = – 6.92*D* (IB3).

**Table 3:** Total Dipole Moment (μ) and Its Components (μ_*i*_), for the Isolated and Embedded Molecules for IB1, IB2, and IB3 Molecular Systems

		μ (D)	μ_*x*_ (*D*)	μ_*y*_ (*D*)	μ_*z*_ (*D*)
IB1	isolated	4.31	1.40	1.21	3.89
	embedded	5.62	1.58	–1.61	**5.15**
					
IB2	isolated	4.04	2.68	0.23	–3.01
	embedded	4.34	**3.21**	–0.04	–2.93
					
IB3	isolated	6.07	1.28	–5.34	2.59
	embedded	8.02	1.74	**-6.92**	3.68

[Table tbl4] shows the static and dynamic (ω = 0.043 a.u and ω = 0.085a.u.) results for the linear and nonlinear electro-optical parameters: ⟨α­(−ω; ω)⟩, *n*(ω), ⟨γ­(−ω; ω,0.0)⟩, ⟨γ­(−ω; ω, ω, −ω)⟩, and χ^(3)^(−ω; ω, ω, −ω)) for IB1, IB2, and IB3 compounds in the crystalline phase. All parameters’ values increase with the increasing of the electric field frequency; this effect is smaller for the linear parameters (less than 7%) and greater for the nonlinear optical parameters ⟨γ­(−ω; ω,0.0)⟩ values, which increase ∼42% with the increase in the electric field frequency. Both the compounds, IB1 and IB2, have one bromine atom attached at carbon atom C_4_, but they differ by the position of the other Br, attached at C_5_ and C_6_, respectively; however, IB3 has the bromine atom attached at C_5_ and C_6_. As we can see from Table 4, the ⟨γ­(−ω; ω,0,0)⟩ values for IB1 and IB2 are similar, and greater than IB3, but the χ^(3)^ values for IB2 and IB3 are greater than for IB1, due to the smaller volume of these compounds, as shown in Table 1.

**Table 4:** Static and Dynamic Results for the Parameters: ⟨α­(−ω; ω)⟩, *n*(ω), ⟨γ­(−ω; ω,0.0)⟩, ⟨γ­(−ω; ω, ω, −ω)⟩, and χ^(3)^ (−ω; ω, ω,−ω)), for IB1, IB2, and IB3 Embedded Molecules

	ω (a.u.)	⟨α(−ω; ω)⟩ (10^–24^ esu)	*n* (ω)	⟨γ(− ω; ω,0,0)⟩ (10^–36^ esu)	⟨γ(− ω; ω, ω, – ω)⟩ (10^–36^ esu)	χ^(3)^(− ω; ω, ω, – ω) (10^–22^ (m/V)^2^)
						
IB1	0.000	23.66	1.67	19.08	19.08	64.21
	0.043	24.00	1.68	20.59	22.09	76.94
	0.085	25.19	1.72	26.84	34.60	136.13
						
IB2	0.000	23.55	1.67	19.35	19.35	67.47
	0.043	23.89	1.69	20.96	22.56	81.43
	0.085	25.07	1.73	27.75	36.16	147.69
						
IB3	0.000	23.75	1.74	18.04	18.04	79.06
	0.043	24.10	1.75	19.43	20.83	94.92
	0.085	25.31	1.80	25.47	32.90	172.65

The χ(3) values of IB1–IB3 (136–173 × 10^–22^ m^2^ V^–2^) are higher than those of 4-methylsulfanyl chalcones (2–2.4 × 10^–22^ m^2^ V^–2^), hydrazone 4MNA (10 × 10^–22^ m^2^ V^–2^), and CTDCP (16 × 10^–22^ m^2^ V^–2^) and approach the best value reported for 3MPNP (277 × 10^–22^ m^2^ V^–2^). These values are very significant when compared with the experimental results obtained for organic compounds from the literature, shown in Table 5. Therefore, the IB1, IB2, and IB3 crystals have the potential to be studied as a nonlinear optical material.

**Table 5:** Theoretical Results for χ^(3)^, at 532 nm, for the IB1, IB2, and IB3^a^

organic crystals	$\chi^{\left(3\right)}\left(10^{-22}{\left(\frac{m}{V}\right)}^{2}\right)$	origin
IB1 (present work)	136.13	*calc* ^b^
IB2 (present work)	147.69	*calc*
IB3 (present work)	172.65	*calc*
2(*E*)-3-(3-methylphenyl)-1-(4-nitrophenyl)prop-2-en-1-one (3MPNP) (3NPMP)^49^	277.10	*exp.*
1-(5-chlorothiophen-2-yl)-3-(2,3-dichlorophenyl)prop-2-en-1-one (CTDCP)^50^	16.21	*exp.*
2-(4-methylphenoxy)-N0-[(1*E*)-(4-nitrophenyl)methylene]acetohydrazide (4MNA)^51^	10.24	*exp.*
1-(4-aminophenyl)-3-(3,4,5-trimethoxyphenyl)prop-2-en-1-one (APTP)^52^	8.70	*exp.*
(2*E*)-3-[4-(methylsulfanyl)phenyl]-1-(4-nitrophenyl)prop-2-en-1-one (4N4MSP)^53^	2.37	*exp.*
(2*E*)-1-(4-bromophenyl)-3-[4-methylsulfanyl)phenyl]prop-2-en-1-one (4Br4MSP)^53^	2.30	*exp.*
(2*E*)-1-(3-bromophenyl)-3-[4 (methylsulfanyl) phenyl]prop-2-en-1-one (3Br4MSP)^53,54^	1.99	*exp.*

a Some experimental results for other organic compounds are shown for comparison.

b Calc. = calculated in this work; exp. = experimental literature value.

The results presented in Table 5 confirm the relationship between the HOMO–LUMO gap and the NLO properties of IB1, IB2, and IB3. It is well-known that a smaller HOMO–LUMO gap can enhance the polarizability of a molecule, facilitating greater electronic charge transfer when exposed to an external electric field. This increased polarizability typically leads to stronger third-order nonlinear optical responses, such as higher values of χ^(3)^. As observed, IB3, which has the smallest HOMO–LUMO gap, also exhibits the highest χ^(3)^ value among the compounds studied. This trend aligns with the theoretical understanding that a reduced energy gap correlates with better NLO properties. These results corroborate prior research, which highlights the accuracy of DFT-based predictions for third-order nonlinear susceptibilities in organic systems.^47^ Therefore, IB1, IB2, and IB3, particularly IB3, show significant potential for applications in nonlinear optical devices, as the smaller energy gap directly contributes to their superior NLO behavior.

## Conclusions

The molecular structures of these compounds were determined by single-crystal X-ray diffraction. The variations in bromine positioning produce small changes in bond lengths, angles, and torsional dynamics, maintaining the generally flat geometry characteristic of aromatic compounds, which has significant implications for the electronic structure and intermolecular interactions. The positioning of bromine atoms also affects the molecular geometry, particularly the bond angles and dihedral angles, which are crucial for electronic conjugation and delocalization. In IB3, the bromine atoms are at adjacent positions (C5 and C6), causing significant deviations in bond angles within the benzene ring. The increased C6–C1–C7 bond angle (124.6°) reflects steric repulsion, leading to an expanded ring structure. Additionally, the methoxy group at C3 in IB3 exhibits a C3–O3–C9 angle of 117.1°, suggesting reduced steric hindrance and allowing the methoxy group to adopt a conformation closer to planarity. The values of the reactivity descriptors, calculated from the energies of the frontier molecular orbitals, were used for a comparative analysis of the antioxidant activity of the compounds. The topological parameters obtained from QTAIM indicate that the electron density (ρ­(*r*)) values in the internuclear regions of intermolecular interactions are very low (ρ­(*r*) < 0.1 au), with positive Laplacian values ∇^2^ρ­(*r*) > 0, characteristic of closed-shell interactions. This suggests that the electron density is depleted at the BCPs, affecting the stability and reactivity of the molecule. In the crystalline arrangements of IB1 and IB2, dimers are formed through C7–H···O1 and C8–H···O1 interactions. The presence of bromine atoms near the carbonyl group influences these interactions. For instance, in IB2, the proximity of a bromine atom to the carbonyl group causes a decrease in the charge density at the BCPs due to an inductive effect, leading to variations in BE and IE. These subtle differences in intermolecular interactions contribute to the overall stability of the crystal structures and influence the electronic properties relevant to NLO behavior.

The third-order nonlinear properties of three derivatives, namely, IB1, IB2, and IB3 crystals, were studied at the DFT/CAM-B3LYP/aug-*cc*-pVTZ level. The Supermolecule approach was used to simulate the crystalline environment of the compounds with 11 × 11 × 11 unit cells. The crystalline environment polarization increased the total dipole moment by 30.4% (IB1), 7.4% (IB2), and 32.1% (IB3). Furthermore, the average linear polarizability (⟨α­(−ω; ω)⟩), the refractive index, the second hyperpolarizabilities, ⟨γ­(−ω; ω,0.0)⟩ and ⟨γ­(−ω; ω, ω, −ω)⟩, and the third-order nonlinear susceptibility, χ^(3)^(−ω; ω, ω, – ω), for IB1, IB2, and IB3 in crystalline phase were calculated. The nonlinear parameters presented an increase of ∼40% with the increase in the electric field frequencies. The DFT results for the χ^(3)^ values at 532 nm (ω = 0.085 *a*.*u*.) are 136.13 × 10^–22^ (m/V)^2^, 147.69 × 10^–22^ (m/V)^2^, and 172.65 × 10^–22^, for IB1, IB2, and IB3, respectively. These results are lower than for 3NPMP^49,55^ but more than eight times the experimental results for other organic crystals presented in Table 5. Therefore, these crystals are a promising material for use in nonlinear optical devices. Given the promising nonlinear optical properties of IB1, IB2, and IB3, future research could explore their application in real-world photonic devices. Further experimental validation of the theoretical χ^(3)^ values under various environmental conditions, as well as the exploration of additional chemical modifications, could enhance their functionality.

## Supplementary Material



## Notes

Detailed experimental procedures, synthetic routes, characterization data (NMR, FTIR), crystallographic information, computational details, additional spectra, and tables (bond lengths, angles, and QTAIM analyses) (PDF)
