# Supplementary material for: Exploring the NLO Properties of Brominated Dimethoxybenzaldehydes: From Synthesis to Molecular Modeling
Source: J Phys Chem A. 2025 Aug 22;129(35):8148–59. doi: 10.1021/acs.jpca.5c04332 (PMC12415829; doi:10.1021/acs.jpca.5c04332)
Supplement: Supplementary file 1 [file jp5c04332_si_001.pdf]

Supplementary Information

Exploring the NLO properties of brominated dimethoxybenzaldehydes: from synthesis to molecular modeling

Clodoaldo Valverde[1,2], Igor D. Borges[2,3], Marco A. Prazeres[3], Antônio S.N. Aguiar[2,3], Angelica Navarrete[4], Gerardo Aguirre[4], Francisco A.P. Osório[5] and Hamilton B. Napolitano[2]

[1] Universidade Paulista - UNIP, 74845-090, Goiânia, GO, Brazil.
[2] Grupo de Química Teórica e Estrutural de Anápolis, Universidade Estadual de Goiás, Anápolis, GO, Brazil
[3] Laboratório de Novos Materiais, Universidade Evangélica de Goiás, Anápolis, GO, Brazil.
[4] Instituto Tecnológico de Tijuana - Centro de Graduados e Investigación en Química, Tijuana, BC, México.
[5] Instituto de Física, Universidade Federal de Goiás, 74.690-900, Goiânia, GO, Brazil.

Table of contents

1. **General Methods** S3
2. **Experimental and characterization details** S3
   2.1. **Synthesis of 4,5-dibromo-2,3-dimethoxybenzaldehyde (6)** S3
   2.1.1. Preparation of 2-formyl-6-methoxyphenyl 4-methylbenzenesulfonate (**2**) S3
   2.1.2. Preparation of 2-hydroxy-3-methoxy-4-nitrobenzaldehyde (**3**) S3
   2.1.3. Preparation of 2,3-dimethoxy-4-nitrobenzaldehyde (**4**) S4
   2.1.4. Preparation of 4-amino-2,3-dimethoxybenzaldehyde (**5**) S5
   2.1.5. Preparation of 4,5-dibromo-2,3-dimethoxybenzaldehyde (**6**) S5
   2.2. **Synthesis of 2,3-dibromo-5,6-dimethoxybenzaldehyde (9)** S5
   2.2.1. Preparation of 2,3-dibromo-6-hydroxy-5-methoxybenzaldehyde (**8**) S5
   2.2.2. Preparation of 2,3-dibromo-5,6-dimethoxybenzaldehyde (**9**) S6
   2.3. **Synthesis of 4,6-dibromo-2,3-dimethoxybenzaldehyde (13)** S6
   2.3.1. Preparation of 3-hydroxy-2-methoxybenzaldehyde (**11**) S6
   2.3.2. Preparation of 4,6-dibromo-3-hydroxy-2-methoxybenzaldehyde (**12**) S6
   2.3.3. Preparation of 4,6-dibromo-2,3-dimethoxybenzaldehyde (**13**) S7

Figure S1. ^{1}H NMR spectrum of 2-formyl-6-methoxyphenyl 4-methylbenzenesulfonate (**2**) S8
Figure S2. ^{13}C NMR spectrum of 2-formyl-6-methoxyphenyl 4-methylbenzenesulfonate (**2**) S9
Figure S3. ^{1}H NMR spectrum of 2-hydroxy-3-methoxy-4-nitrobenzaldehyde (**3**) S10
Figure S4. ^{13}C NMR spectrum of 2-hydroxy-3-methoxy-4-nitrobenzaldehyde (**3**) S11
Figure S5. ^{1}H NMR spectrum of 2,3-dimethoxy-4-nitrobenzaldehyde (**4**) S12
Figure S6. ^{13}C NMR spectrum of 2,3-dimethoxy-4-nitrobenzaldehyde (**4**) S13

Figure S7. ^{1}H NMR spectrum of 4-amino-2,3-dimethoxybenzaldehyde (**5**) S14

Figure S8. ^{13}C NMR spectrum of 4-amino-2,3-dimethoxybenzaldehyde (**5**) S15

Figure S9. ^{1}H NMR spectrum of 4,5-dibromo-2,3-dimethoxybenzaldehyde (**6**) S16

Figure S10. ^{13}C NMR spectrum of 4,5-dibromo-2,3-dimethoxybenzaldehyde (**6**) S17

Figure S11. ^{1}H NMR spectrum of 2,3-dibromo-6-hydroxy-5-methoxybenzaldehyde (**8**) S18

Figure S12. ^{13}C NMR spectrum of 2,3-dibromo-6-hydroxy-5-methoxybenzaldehyde (**8**) S19

Figure S13. ^{1}H NMR spectrum of 2,3-dibromo-5,6-dimethoxybenzaldehyde (**9**) S20

Figure S14. ^{13}C NMR spectrum of 2,3-dibromo-5,6-dimethoxybenzaldehyde (**9**) S21

Figure S15. ^{1}H NMR spectrum of 3-hydroxy-2-methoxybenzaldehyde (**11**) S22

Figure S16. ^{13}C NMR spectrum of 3-hydroxy-2-methoxybenzaldehyde (**11**) S23

Figure S17. ^{1}H NMR spectrum of 4,6-dibromo-3-hydroxy-2-methoxybenzaldehyde (**12**) S24

Figure S18. ^{13}C NMR spectrum of 4,6-dibromo-3-hydroxy-2-methoxybenzaldehyde (**12**) S25

Figure S19. ^{1}H NMR spectrum of 4,6-dibromo-2,3-dimethoxybenzaldehyde (**13**) S26

Figure S20. ^{13}C NMR spectrum of 4,6-dibromo-2,3-dimethoxybenzaldehyde (**13**) S27

Figure S21. FTIR spectrum of 2-formyl-6-methoxyphenyl 4-methylbenzenesulfonate (**2**) S28

Figure S22. FTIR spectrum of 2-hydroxy-3-methoxy-4-nitrobenzaldehyde (**3**) S29

Figure S23. FTIR spectrum of 2,3-dimethoxy-4-nitrobenzaldehyde (**4**) S30

Figure S24. FTIR spectrum of 4-amino-2,3-dimethoxybenzaldehyde (**5**) S31

Figure S25. FTIR spectrum of 4,5-dibromo-2,3-dimethoxybenzaldehyde (**6**) S32

Figure S26. FTIR spectrum of 2,3-dibromo-6-hydroxy-5-methoxybenzaldehyde (**8**) S33

Figure S27. FTIR spectrum of 2,3-dibromo-5,6-dimethoxybenzaldehyde (**9**) S34

Figure S28. FTIR spectrum of 3-hydroxy-2-methoxybenzaldehyde (**11**) S35

Figure S29. FTIR spectrum of 4,6-dibromo-3-hydroxy-2-methoxybenzaldehyde (**12**) S36

Figure S30. FTIR spectrum of 4,6-dibromo-2,3-dimethoxybenzaldehyde (**13**) S37

Table S1. Relevant experimental and theoretical bond length (Å), bond angles (°) and dihedral angles (°) for DMB's. S38

Table S2. QTAIM topological parameters to the intermolecular interactions observed in IB1, IB2, and IB3 compounds. S39

General Methods

All reagents were purchased in the highest quality available and were used without further purification. ^{1}H and ^{13}C NMR spectra were recorded using a Bruker Avance 400 MHz Spectrometer in $CDCl_3$ relative to tetramethylsilane (TMS) as internal standard. The chemical shifts are expressed in parts per million (ppm, δ) and the coupling constants (*J*) in Hertz (Hz). Infrared spectra were recorded on a FT-IR 1600 spectrophotometer. Melting points were determined on a Fisher-Johns melting point apparatus and were not corrected.

Experimental and characterization details

2.1. Synthesis of 4,5-dibromo-2,3-dimethoxybenzaldehyde (6)

2.1.1. Preparation of 2-formyl-6-methoxyphenyl 4-methylbenzenesulfonate (**2**)

To a solution of *o*-vanillin (**1**) (5.00 g, 32.9 mmol) and sodium hydroxide (2.00 g) in water (25 mL) was added dropwise *p*-toluenesulfonyl chloride (7.51 g. 39.4 mmol) in dichloromethane (50 mL). The mixture was stirred at room temperature for 24 h. The mixture was diluted with water and neutralized with aqueous hydrochloric acid and extracted with dichloromethane (3 × 50 mL). The combined organic layers were washed with saturated solution of sodium hydrogen carbonate and brine. The organic phase was dried over anhydrous sodium sulfate and concentrated. The residue was purified by flash chromatography on silica gel with petroleum ether/ethyl acetate (8:2) to afford 2-formyl-6-methoxyphenyl 4-methylbenzenesulfonate (**2**) as a white solid (9.07 g, 90% yield); mp 97–99 °C; FTIR (ATR): 3090, 2981, 2883, 1690, 1474, 1279, 1147 cm^{-1}; ^{1}H NMR (400 MHz, $CDCl_3$): δ 10.11 (s, 1H), 7.76 (d, *J* = 8.4 Hz, 2H), 7.47 (dd, *J* = 7.8, 1.5 Hz, 1H), 7.32 (m, 3H), 7.10 (dd, *J* = 8.2, 1.4 Hz, 1H), 3.56 (s, 3H), 2.45 (s, 3H); ^{13}C NMR (101 MHz, $CDCl_3$): δ 188.07, 152.67, 145.84, 140.69, 133.02, 131.36, 129.74, 128.71, 127.98, 119.49, 118.09, 56.00, 21.79 ppm.

2.1.2. Preparation of 2-hydroxy-3-methoxy-4-nitrobenzaldehyde (**3**)

To a suspension of 2-formyl-6-methoxyphenyl 4-methylbenzenesulfonate (**2**) (8.90 g, 29.1 mmol) and potassium nitrate (3.23 g, 31.9 mmol) in chloroform (20 mL) trifluoroacetic anhydride (38 mL) was added at 0 °C. The reaction was stirred at 0 °C for 2 h, and then at room temperature overnight. The mixture was diluted carefully with water (150 mL) and extracted with chloroform (3 × 70 mL). The residue was purified by flash chromatography on silica gel with petroleum ether/ethyl acetate (8:2) to obtain the intermediate 6-formyl-2-methoxy-3-nitrophenyl 4-methylbenzenesulfonate (3.52 g, 34% yield) used directly for next step. The intermediate (3.52 g, 10.0 mmol) was solved in methanol (30 mL) and sodium hydroxide (1.51 g, 37.7 mmol) in distilled water (10 mL) was added dropwise maintaining reflux temperature. The reaction was refluxed for 30 min. Distilled water (150 mL) was added and the solution treated with concentrated hydrochloric acid until pH = 3–5, and followed by extraction into dichloromethane (3 × 50 mL). The organic phase was dried over anhydrous sodium sulfate and concentrated. The residue was purified by flash chromatography on silica gel with petroleum ether/ethyl acetate (9:1) to afford 2-hydroxy-3-methoxy-4-nitrobenzaldehyde (**3**) as an orange solid (1.39 g, 70% yield); mp 89–91 °C; FTIR (ATR): 3110, 3055, 2934, 1632, 1501, 1451 cm^{-1}; ^{1}H NMR (400 MHz, $CDCl_3$): δ 11.30 (s, 1H), 9.91 (s, 1H), 7.45 (d, *J* = 8.40 Hz, 1H), 7.30 (d, *J* = 8.30 Hz, 1H), 4.00 (s, 3H); ^{13}C NMR (101 MHz, $CDCl_3$): δ 196.28, 156.54, 148.58, 141.92, 127.86, 123.06, 114.42, 62.12 ppm.

2.1.3. Preparation of 2,3-dimethoxy-4-nitrobenzaldehyde (**4**)

To a solution of 2-hydroxy-3-methoxy-4-nitrobenzaldehyde (**3**) (1.00 g, 5.07 mmol) and potassium carbonate (1.05 g, 7.60 mmol) in dry dimethylformamide (5 mL) methyl iodide (1.08 g, 7.60 mmol) was added and the mixture was stirred for 3 h at room temperature. The reaction mixture was quenched with water (10 mL) and extracted with diethyl ether (3 × 10 mL). The organic phase was dried over anhydrous sodium sulfate and concentrated to afford 2,3-dimethoxy-4-nitrobenzaldehyde **(4)** as a yellow viscous liquid (1.06 g, 98% yield); FTIR (ATR): 3089, 2946, 2869, 1692, 1589, 1525 cm^{-1}; ^{1}H NMR (400 MHz, $CDCl_3$): δ 10.35 (s, 1H), 7.59 (d, *J* = 8.5 Hz, 1H), 7.47 (d, *J* = 8.5 Hz, 1H), 4.05 (s, 3H), 3.99 (s, 3H); ^{13}C NMR (101 MHz, $CDCl_3$): δ 188.22, 157.52, 148.81, 147.15, 132.67, 122.86, 119.21, 62.69, 62.28 ppm.

2.1.4. Preparation of 4-amino-2,3-dimethoxybenzaldehyde (**5**)

To a solution of 2,3-dimethoxy-4-nitrobenzaldehyde (**4**) (1.06 g, 5.02 mmol) in ethanol/water (20:2 mL) Fe powder (2.80 g, 50.2 mmol) and concentrated hydrochloric acid (0.08 g) was added. The reaction mixture was refluxed and stirred vigorously for 2 h and then cooled to room temperature. The solvent was removed under reduced pressure and the residue was filtered through a plug of silica gel to afford 4-amino-2,3-dimethoxybenzaldehyde (**5**) as a yellow viscous liquid (0.89 g, 98% yield); FTIR (ATR): 3336, 2963, 2929, 2874, 1463 cm^{-1}; ^{1}H NMR (400 MHz, $CDCl_3$): δ 10.08 (s, H), 7.42 (d, J = 8.5 Hz, 1H), 6.48 (d, J = 8.5 Hz, 1H), 4.53 (s, 2H), 3.96 (s, 3H), 3.84 (s, 3H); ^{13}C NMR (101 MHz, $CDCl_3$): δ 188.15, 156.89, 147.93, 138.20, 125.34, 120.68, 110.46, 61.96, 60.06 ppm.

2.1.5. Preparation of 4,5-dibromo-2,3-dimethoxybenzaldehyde (**6**)

To a solution of 4-amino-2,3-dimethoxybenzaldehyde (**5**) (0.50 g, 2.76 mmol) in acetonitrile (25 mL) copper(II) bromide (3.08 g, 13.8 mmol) and *tert*-butyl nitrite (0.43 g, 4.16 mmol) was added. The reaction was heated at 50 °C and stirred for 1 h and then cooled to room temperature. The mixture was diluted with saturated solution of sodium hydrogen carbonate and extracted with ethyl acetate (3 × 30 mL). The combined organic phase was dried over anhydrous sodium sulfate and concentrated. The residue was purified by flash chromatography on silica gel with petroleum ether/ethyl acetate (9:1) to afford 4,5-dibromo-2,3-dimethoxybenzaldehyde (**6**) as a pale yellow solid (0.86 g, 96% yield); mp 96–97 °C; FTIR (ATR): 2938, 2877, 1682, 1559, 1460 cm^{-1}; ^{1}H NMR (400 MHz, $CDCl_3$): δ 10.27 (s, 1H), 7.84 (s,1H), 4.02 (s, 3H), 3.91 (s, 3H); ^{13}C NMR (101 MHz, $CDCl_3$): δ 187.84, 155.45, 152.61, 129.86, 129.06, 127.13, 120.59, 62.55, 60.94 ppm.

2.2. Synthesis of 2,3-dibromo-5,6-dimethoxybenzaldehyde (9)

2.2.1. Preparation of 2,3-dibromo-6-hydroxy-5-methoxybenzaldehyde (**8**)

To a solution of 6-bromo-2-hydroxy-3-methoxybenzaldehyde (**7**) (1.00 g, 4.33 mmol) in dry tetrahydrofuran (50 mL) *N*-bromosuccinimide (0.847 g, 4.76 mmol) was added and the reaction was stirred for 30 min at room temperature. The mixture was quenched with saturated solution of sodium thiosulfate and extracted with ethyl acetate

(3 × 30 mL). The combined organic phase was dried over anhydrous sodium sulfate and concentrated. The residue was purified by flash chromatography on silica gel with petroleum ether/dichloromethane (1:1) to afford 2,3-dibromo-6-hydroxy-5-methoxybenzaldehyde (**8**) as a yellow solid (1.14 g, 85% yield); mp 144–145 °C; FTIR (ATR): 3080, 3007, 2931, 2881, 1634, 1456 cm^{-1}; ^1H NMR (400 MHz, $CDCl_3$): δ 12.44 (s,1H), 10.31 (s,1H), 7.22 (s, 1H), 3.89 (s, 3H); ^{13}C NMR (101 MHz, $CDCl_3$): δ 199.23, 154.09, 148.86, 121.60, 118.70, 118.44, 115.14, 56.70 ppm.

2.2.2. Preparation of 2,3-dibromo-5,6-dimethoxybenzaldehyde (**9**)

The compound 2,3-dibromo-5,6-dimethoxybenzaldehyde (**9**) was synthesized from (**8**) (1.00 g, 3.23 mmol) using the same procedure described for compound (**4**) to obtain (**9**) as a yellow solid (0.994 g, 96% yield); mp 103–104 °C; FTIR (ART): 3066, 2929, 2879, 1691, 1451 cm^{-1}; ^1H NMR (400 MHz, $CDCl_3$): δ 10.19 (s, 1H), 7.32 (s, 1H), 3.89 (s, 3H), 3.88 (s, 3H); ^{13}C NMR (101 MHz, $CDCl_3$): δ 190.23, 153.06, 150.40, 131.39, 121.42, 120.64, 114.74, 62.57, 56.56 ppm.

2.3. Synthesis of 4,6-dibromo-2,3-dimethoxybenzaldehyde (13)

2.3.1. Preparation of 3-hydroxy-2-methoxybenzaldehyde (**11**)

To a solution of 2,3-dihydroxybenzaldehyde (**10**) (6.00 g, 43.4 mmol) and potassium carbonate (6.00 g, 43.4 mmol) in dry dimethylformamide (100 mL) methyl iodide (8.00 g, 56.4 mmol) was added and the mixture was stirred for 20 h at room temperature. The reaction mixture was quenched with water (100 mL) and extracted with diethyl ether (3 × 70 mL). The organic phase was dried over anhydrous sodium sulfate and concentrated. The residue was purified by flash chromatography with petroleum ether/ethyl acetate (9:1) to afford 3-hydroxy-2-methoxybenzaldehyde (**11**) as a pale yellow solid (3.97 g, 60% yield); mp 109–110 °C; FTIR (ATR): 3173, 2956, 2883, 1659, 1573 cm^{-1}; ^1H NMR (400 MHz, $CDCl_3$): δ 10.29 (s, 1H), 7.39 (dd, J = 7.7, 1.7 Hz, 1H), 7.25 (dd, J = 8.0, 1.7 Hz, 1H), 7.15 (t, J = 7.8 Hz, 1H), 6.22 (s, 1H), 3.99 (s, 3H); ^{13}C NMR (101 MHz, $CDCl_3$) δ 189.92, 149.69, 149.54, 129.23, 125.17, 122.22, 121.66, 63.89 ppm.

2.3.2. Preparation of 4,6-dibromo-3-hydroxy-2-methoxybenzaldehyde (**12**)

To a solution of 3-hydroxy-2-methoxybenzaldehyde (**11**) (1.00 g, 6.57 mmol) and sodium acetate (1.67 g, 20.3 mmol) in glacial acetic acid (50 mL) bromine (2.1 g, 13.2 mmol) was added. The mixture was stirred for 1 h at room temperature and then the solvent was removed under reduced pressure. To the residue water was added and extracted with dichloromethane (3 × 50 mL). The organic phase was washed with aqueous solution of sodium carbonate and water. The organic phase was dried over anhydrous sodium sulfate and concentrated to afford 4,6-dibromo-3-hydroxy-2-methoxybenzaldehyde (**12**) as a pale yellow solid (1.96 g, 96% yield); mp 135–136 °C; FTIR (ATR): 3402, 3075, 2955, 2861, 1688, 1558 cm^{-1}; NMR (400 MHz, $CDCl_3$): δ 10.28 (s, 1H), 7.61 (s, 1H), 6.23 (s,1H), 3.96 (s, 3H); ^{13}C NMR (101 MHz, $CDCl_3$): δ 190.10, 148.76, 147.32, 132.44, 126.29, 115.54, 115.07, 63.43 ppm.

2.3.3. Preparation of 4,6-dibromo-2,3-dimethoxybenzaldehyde (**13**)

The compound 4,6-dibromo-2,3-dimethoxybenzaldehyde (**13**) was synthesized from (**12**) (1.00 g, 3.23 mmol) using the same procedure described for compound (**4**) to obtain (**13**) as a pale yellow solid (0.970 g, 93% yield); mp 65–67 °C; FTIR (ATR): 3078, 2941, 2852, 1693, 1536 cm^{-1}; ^{1}H NMR (400 MHz, $CDCl_3$): δ 10.26 (s,1H), 7.64 (s,1H), 3.97 (s, 3H), 3.89 (s, 3H); ^{13}C NMR (101 MHz, $CDCl_3$): δ 189.55, 156.40, 151.16, 133.01, 128.52, 124.27, 118.06, 62.68, 61.05 ppm.

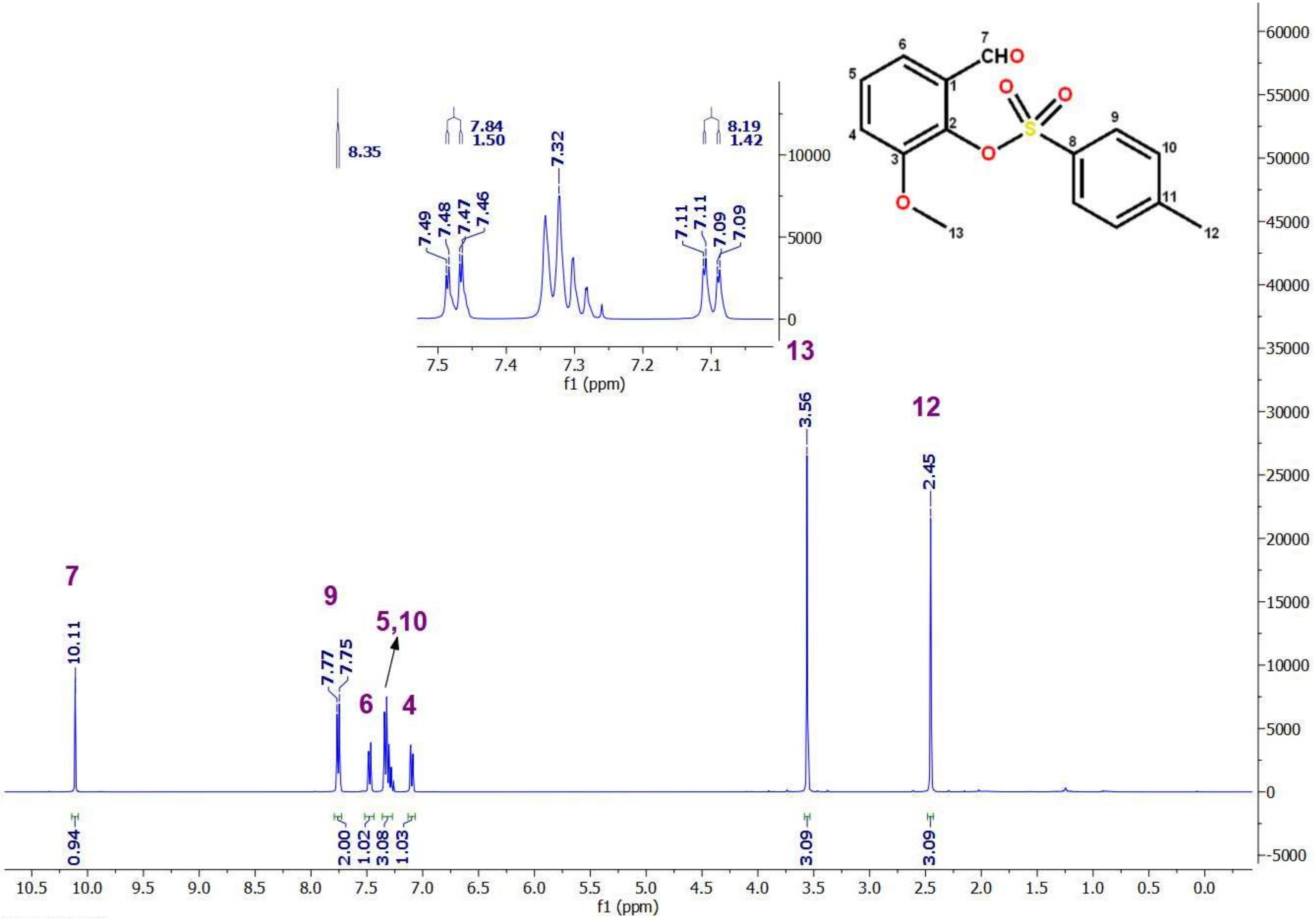

Figure S1. ^{1}H NMR spectrum of 2-formyl-6-methoxyphenyl 4-methylbenzenesulfonate (**2**)

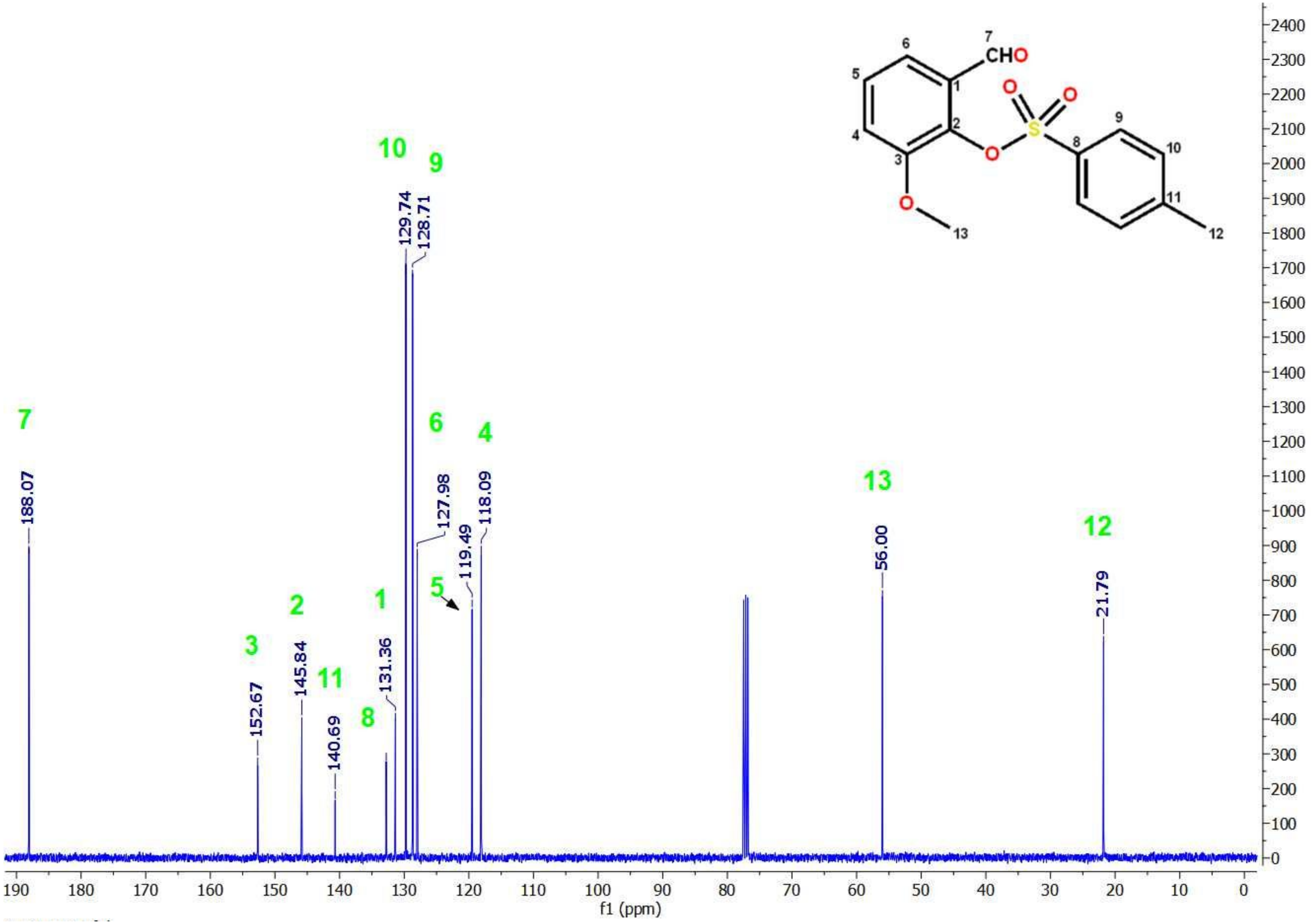

Figure S2. ^{13}C NMR spectrum of 2-formyl-6-methoxyphenyl 4-methylbenzenesulfonate (**2**)

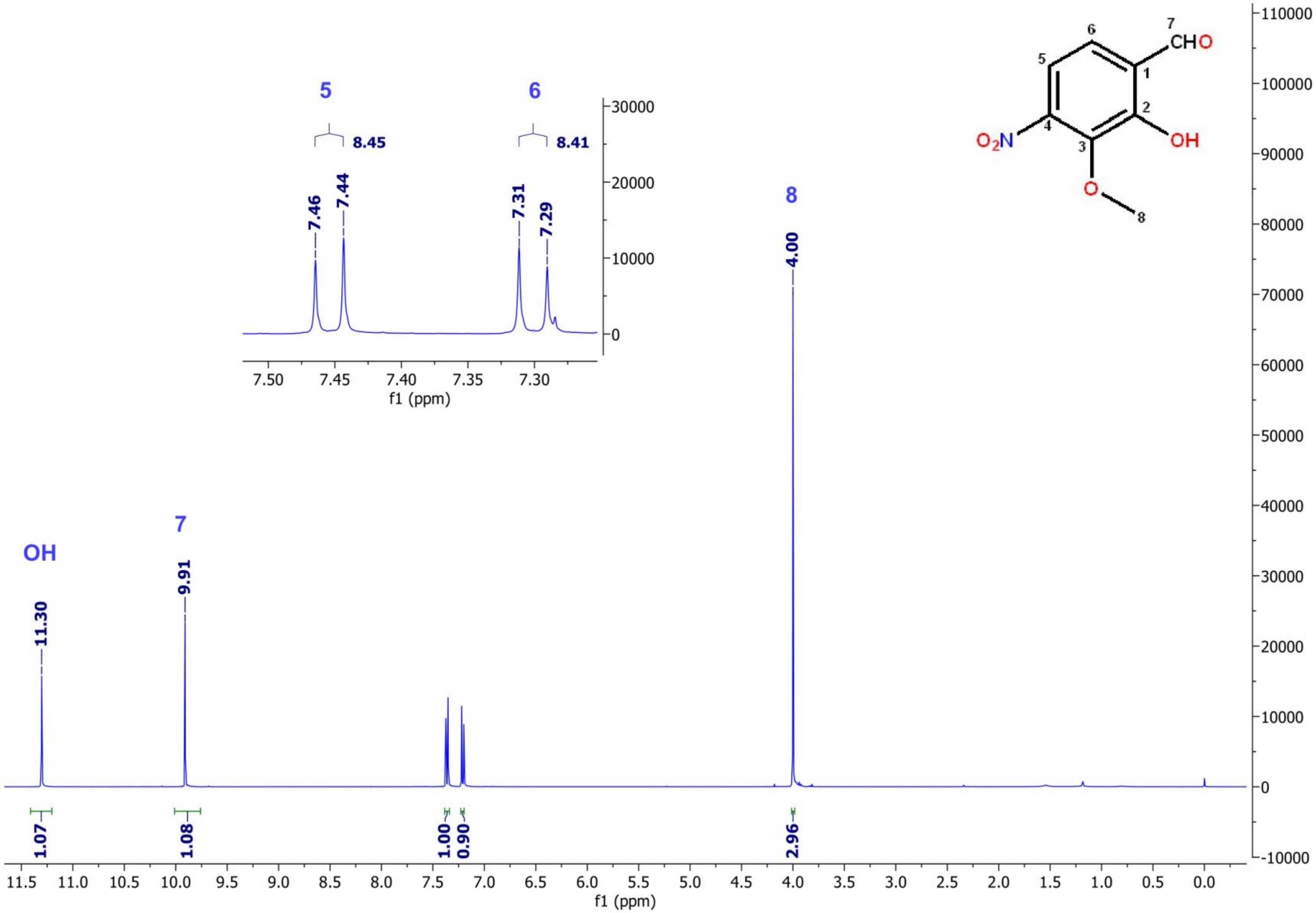

Figure S3. ^{1}H NMR spectrum of 2-hydroxy-3-methoxy-4-nitrobenzaldehyde (**3**)

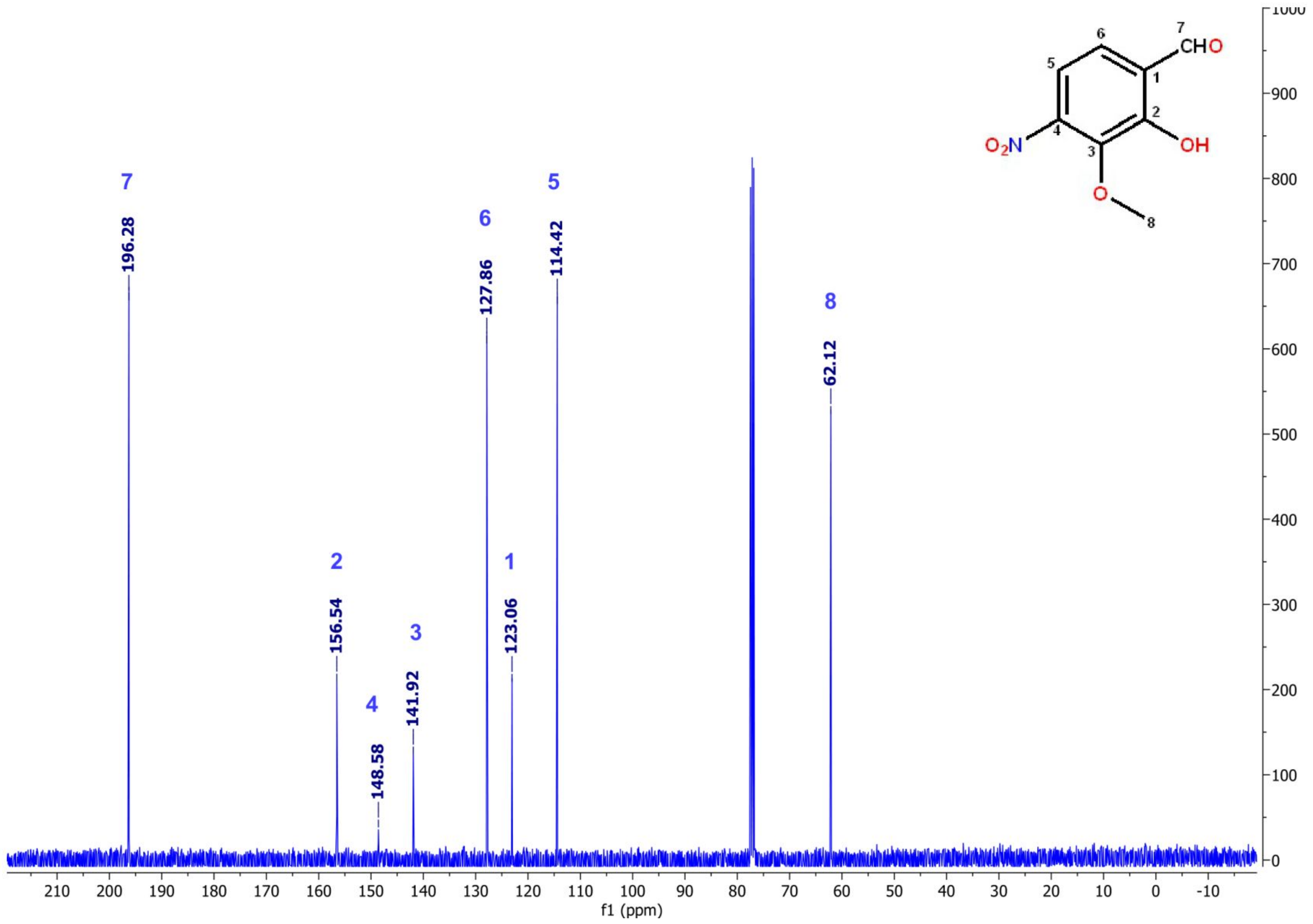

Figure S4. ^{13}C NMR spectrum of 2-hydroxy-3-methoxy-4-nitrobenzaldehyde (**3**)

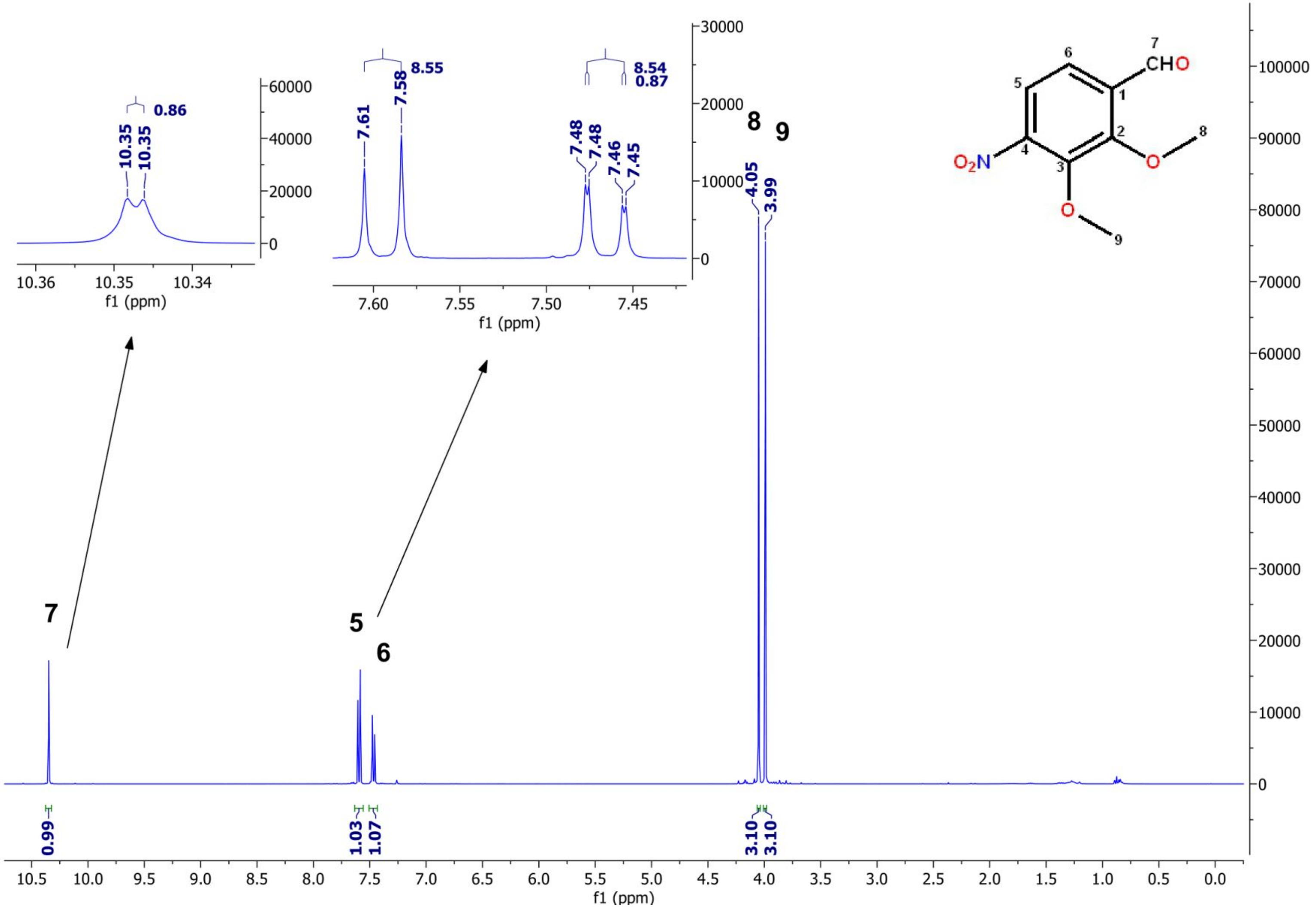

Figure S5. ^{1}H NMR spectrum of 2,3-dimethoxy-4-nitrobenzaldehyde (**4**)

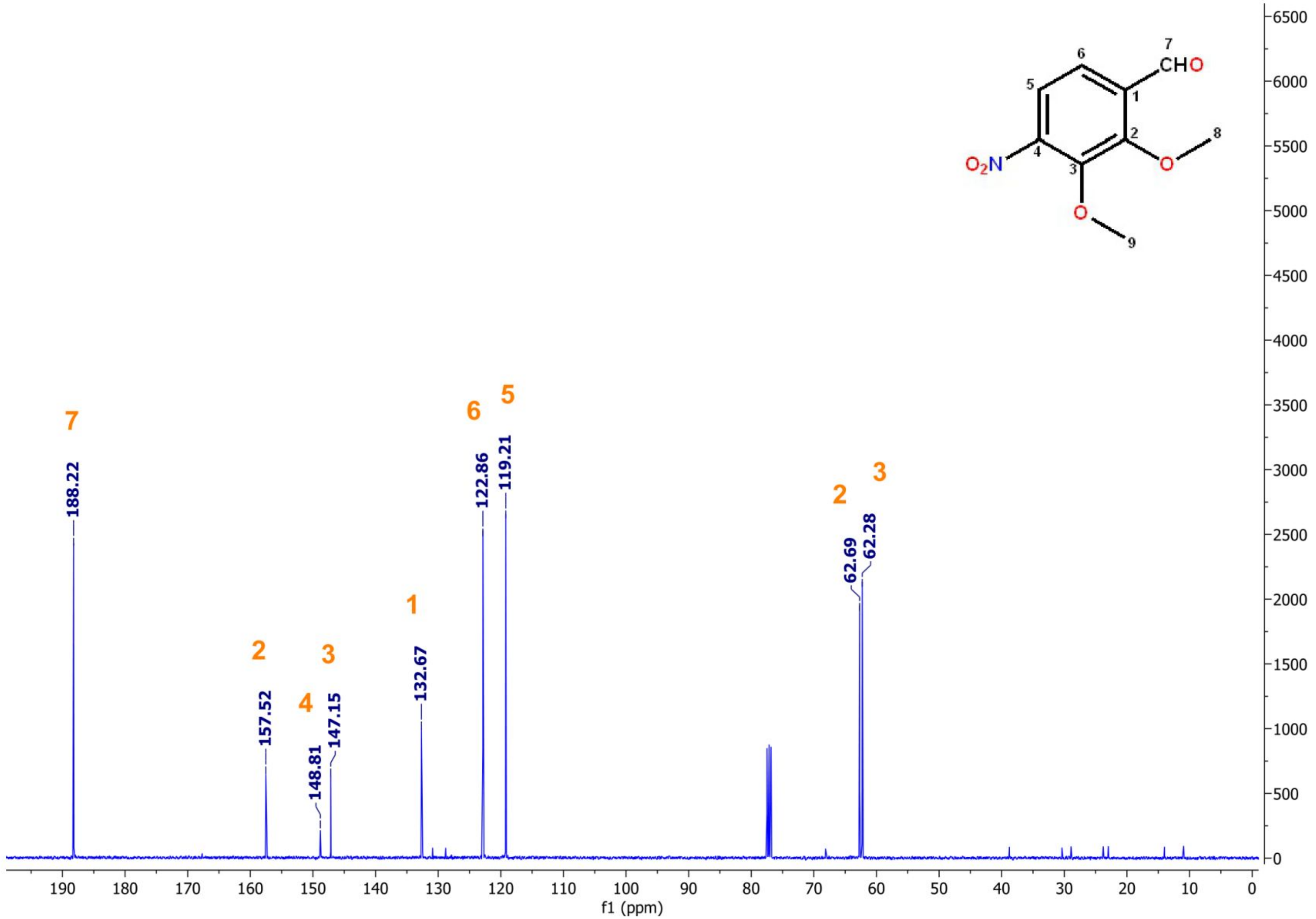

Figure S6. ^{13}C NMR spectrum of 2,3-dimethoxy-4-nitrobenzaldehyde (**4**)

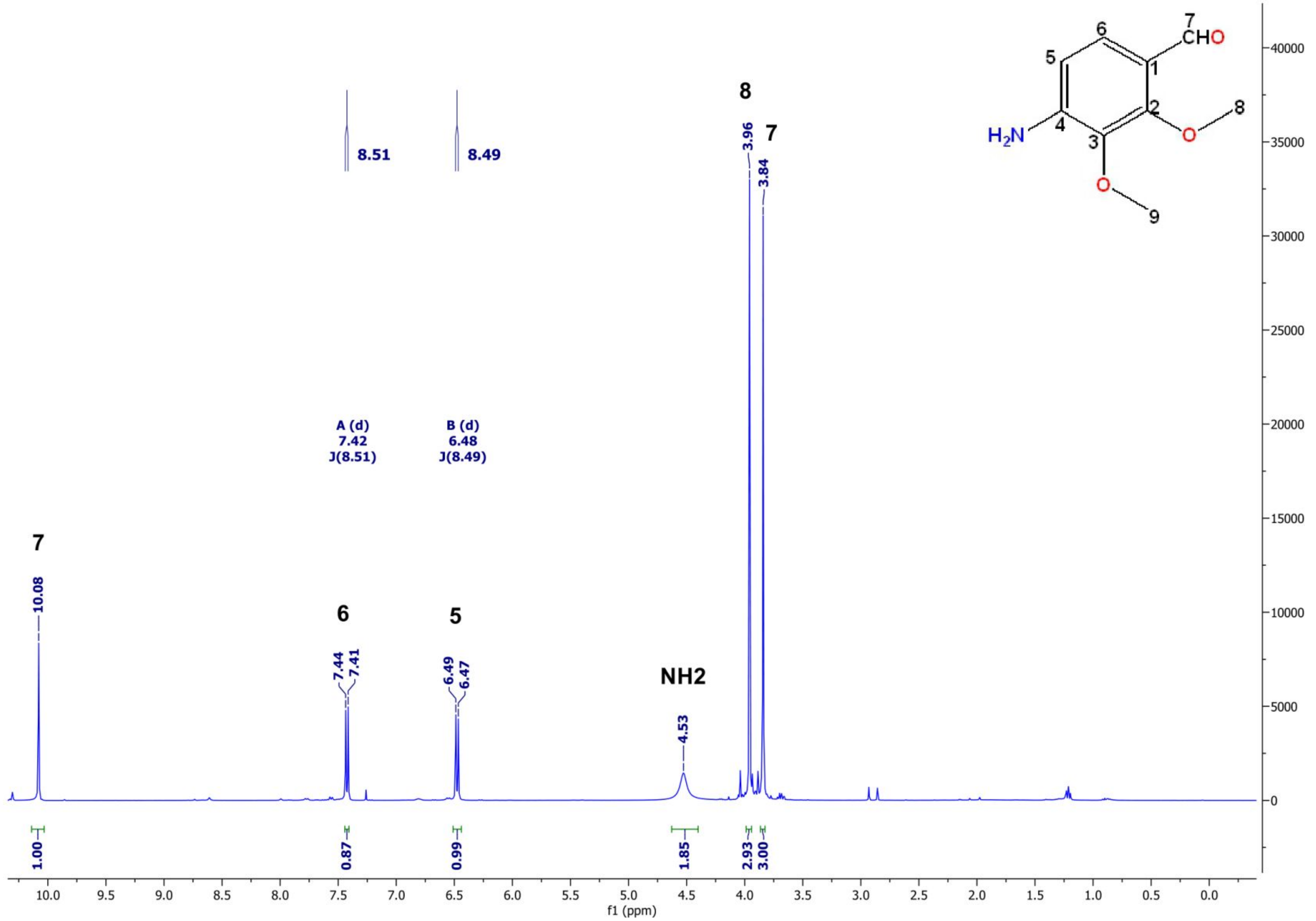

Figure S7. ^{1}H NMR spectrum of 4-amino-2,3-dimethoxybenzaldehyde (**5**)

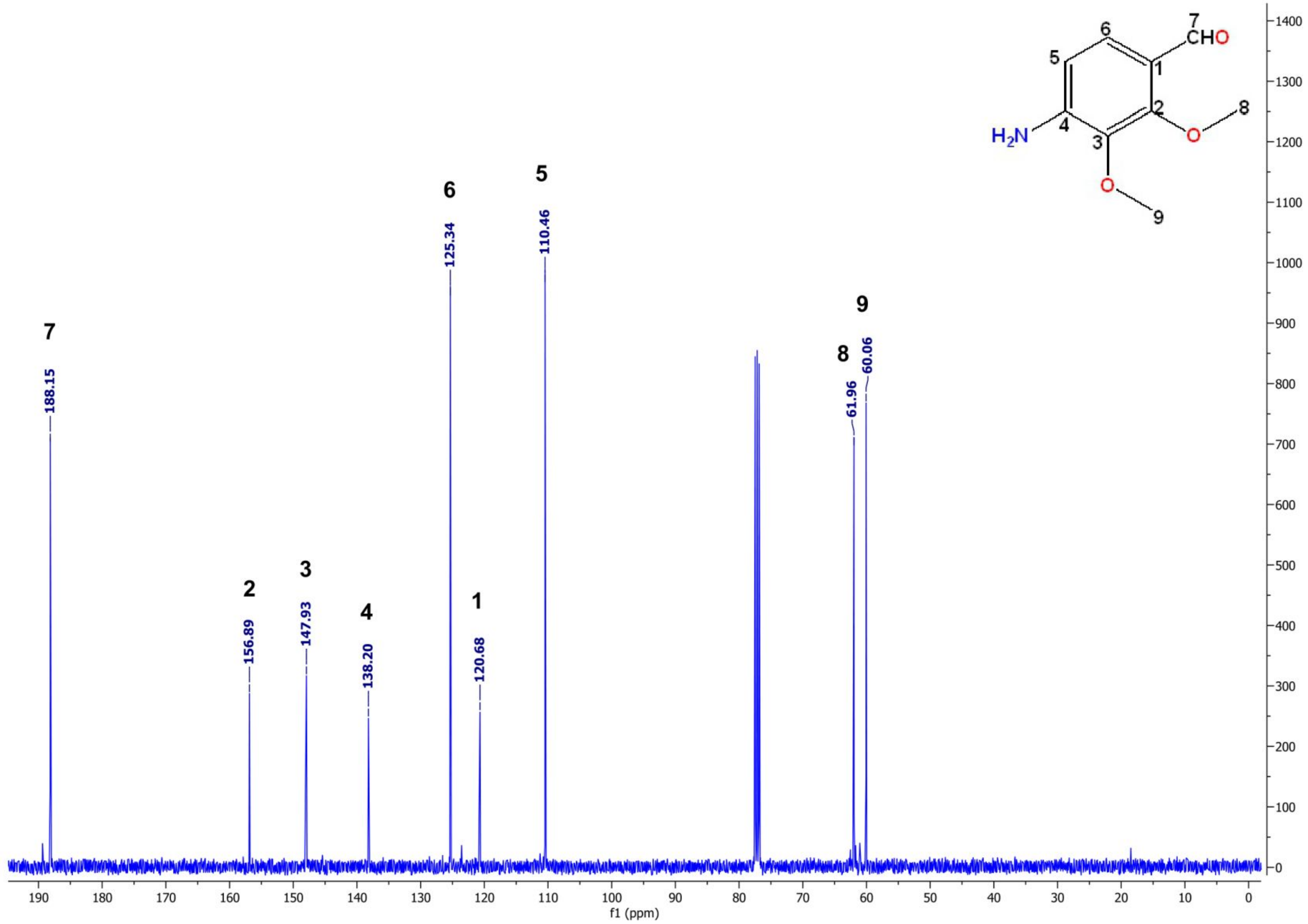

Figure S8. ^{13}C NMR spectrum of 4-amino-2,3-dimethoxybenzaldehyde (**5**)

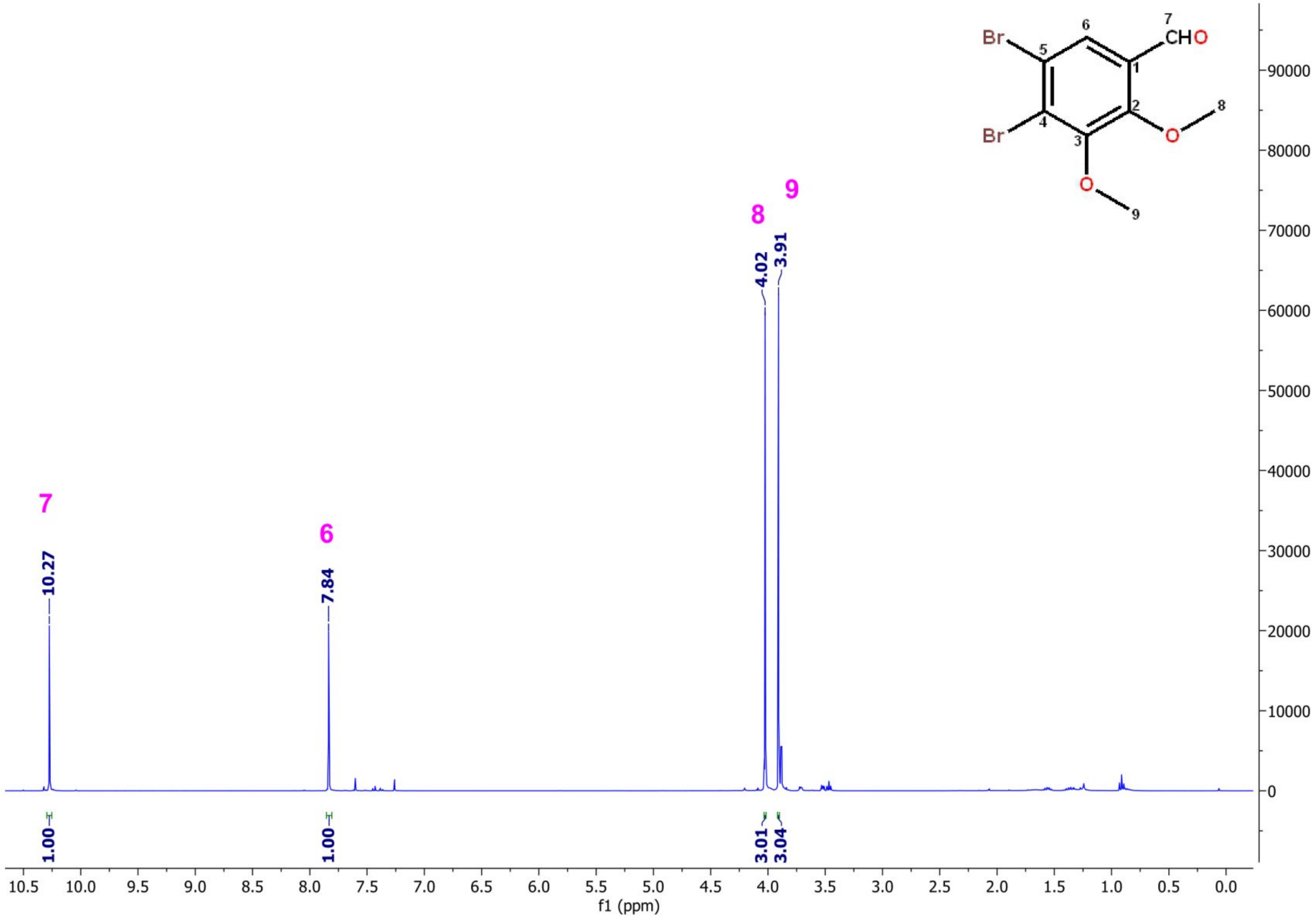

Figure S9. ^{1}H NMR spectrum of 4,5-dibromo-2,3-dimethoxybenzaldehyde (**6**)

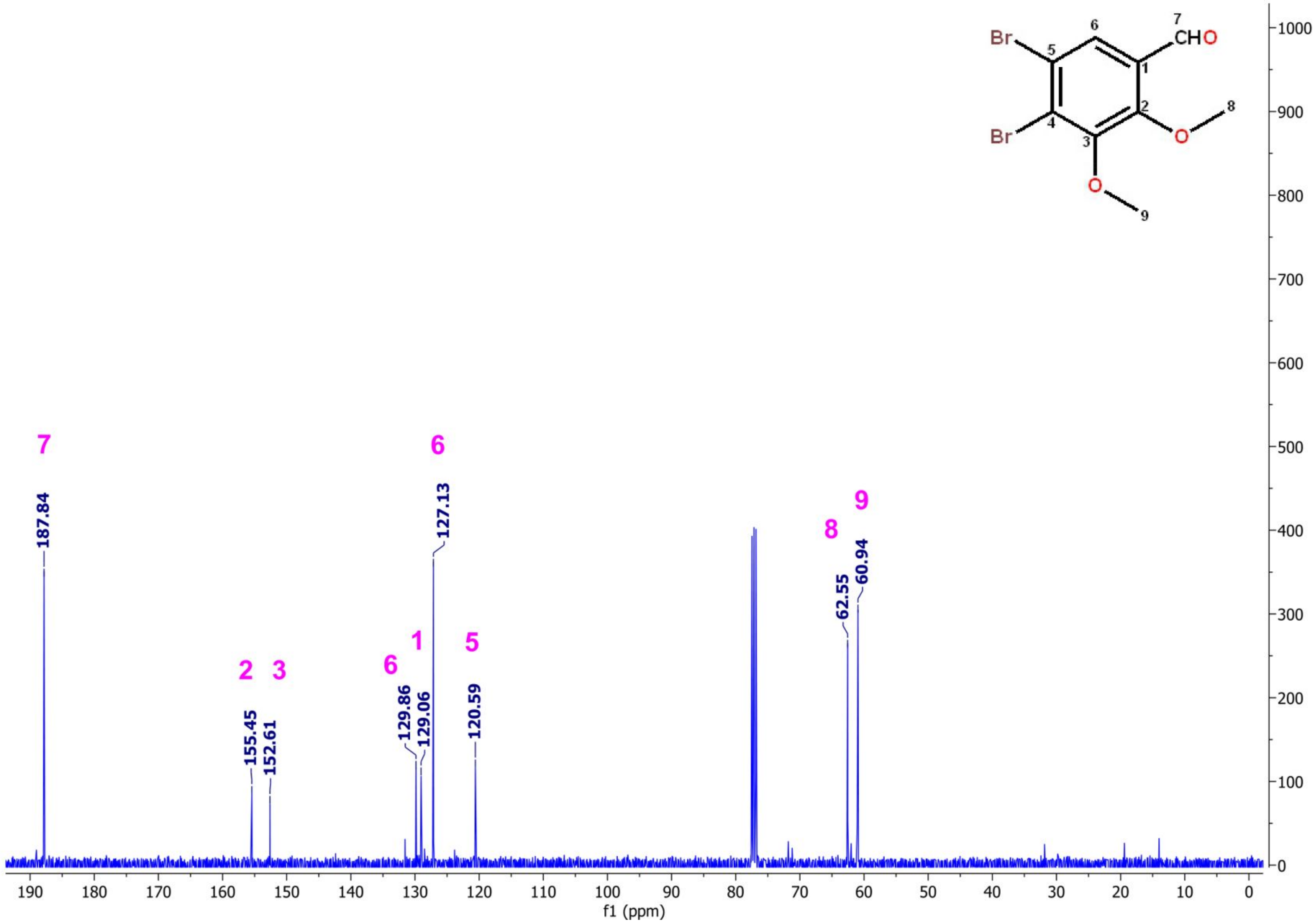

Figure S10. ^{13}C NMR spectrum of 4,5-dibromo-2,3-dimethoxybenzaldehyde (**6**)

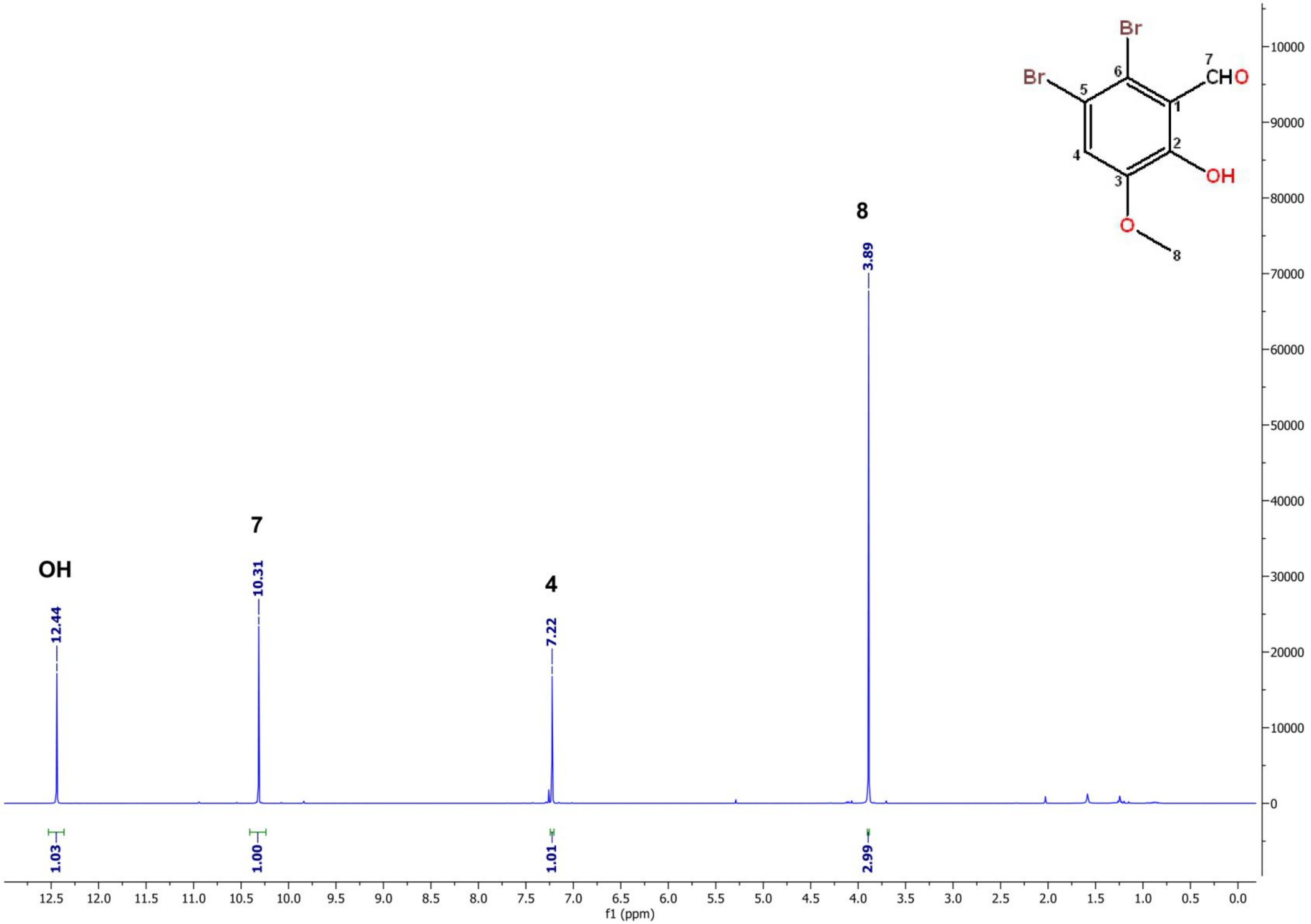

Figure S11. ^{1}H NMR spectrum of 2,3-dibromo-6-hydroxy-5-methoxybenzaldehyde (**8**)

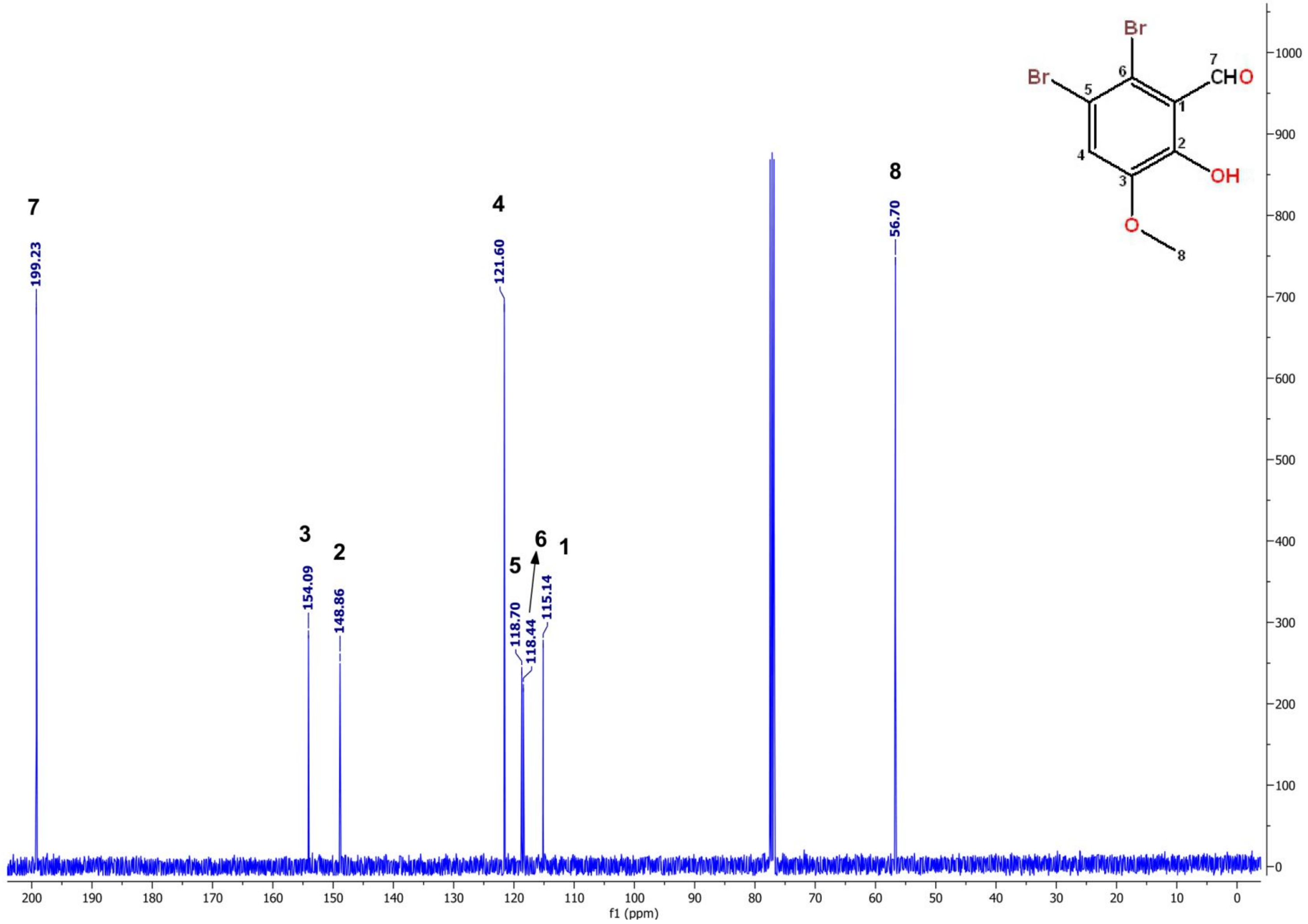

Figure S12. ^{13}C NMR spectrum of 2,3-dibromo-6-hydroxy-5-methoxybenzaldehyde (**8**)

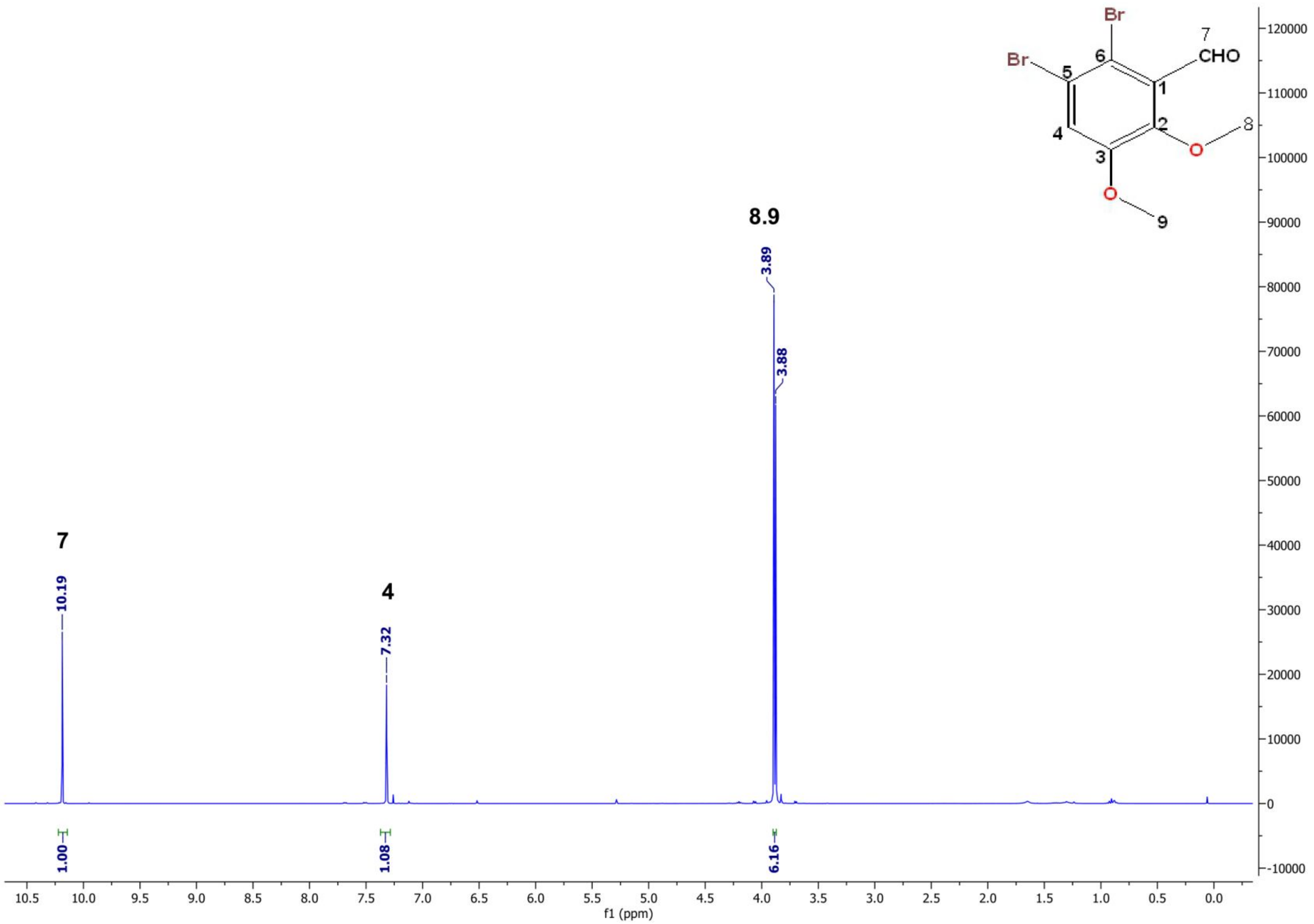

Figure S13. ^{1}H NMR spectrum of 2,3-dibromo-5,6-dimethoxybenzaldehyde (**9**)

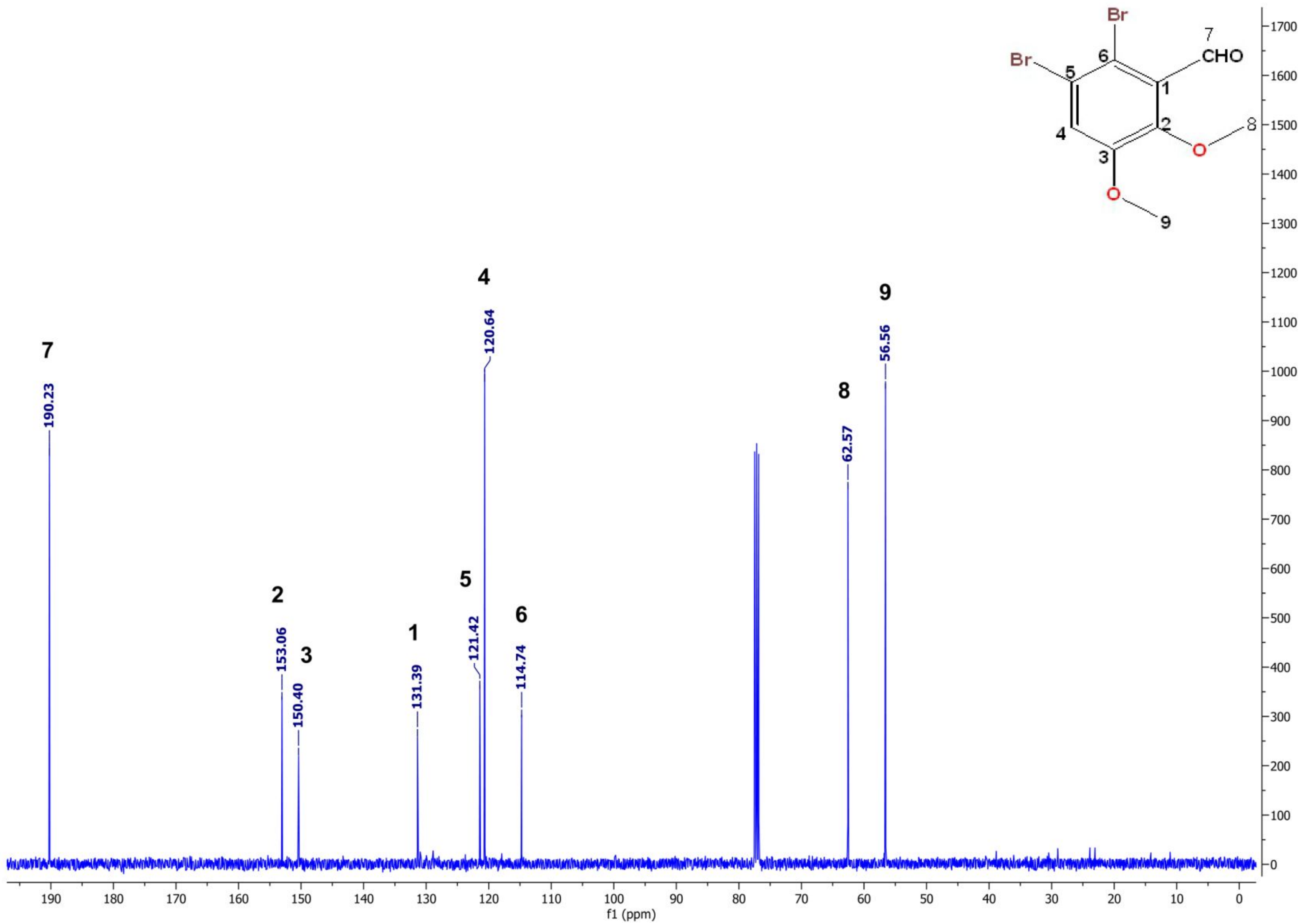

Figure S14. ^{13}C NMR spectrum of 2,3-dibromo-5,6-dimethoxybenzaldehyde (**9**)

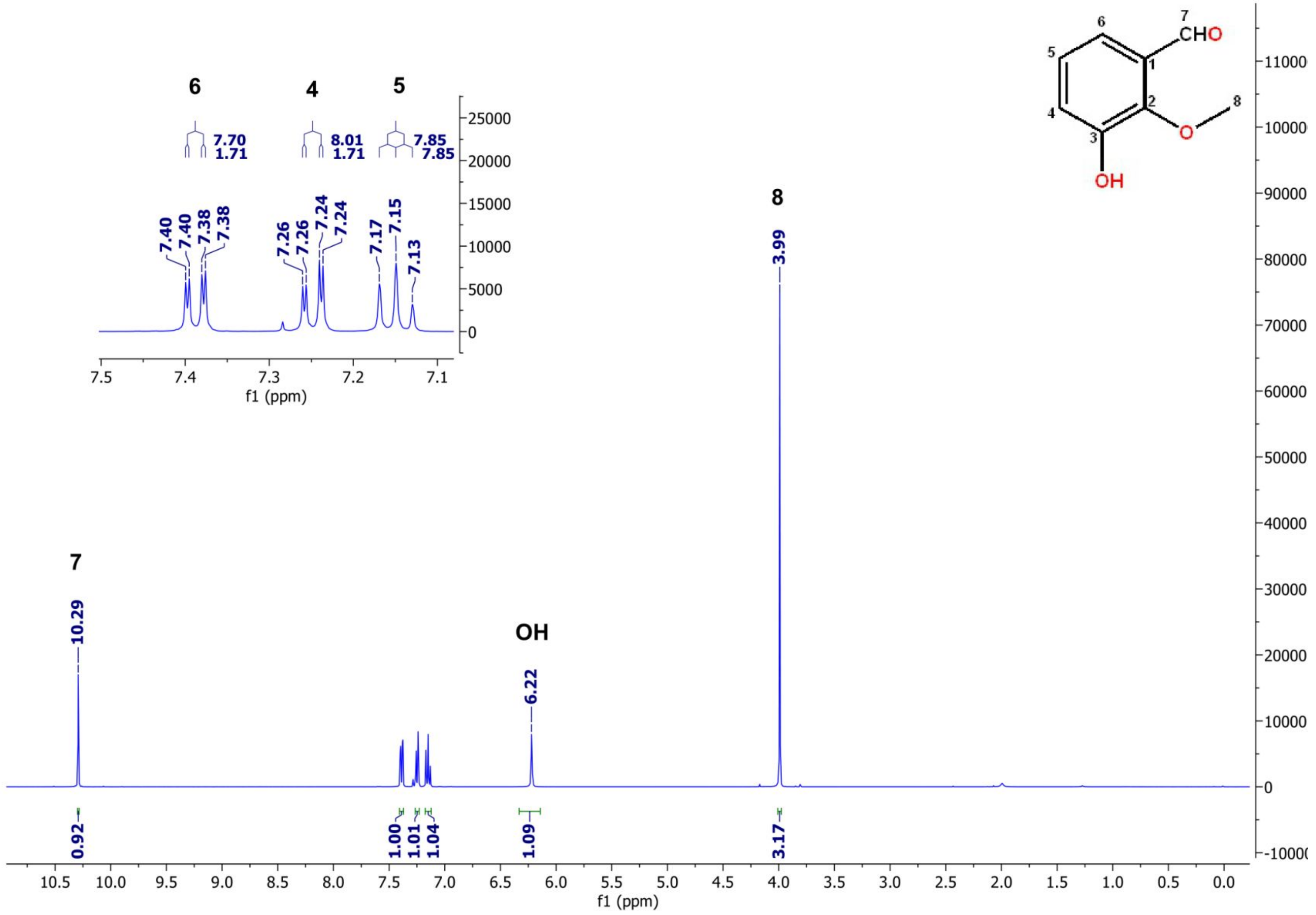

Figure S15. ^{1}H NMR spectrum of 3-hydroxy-2-methoxybenzaldehyde (**11**)

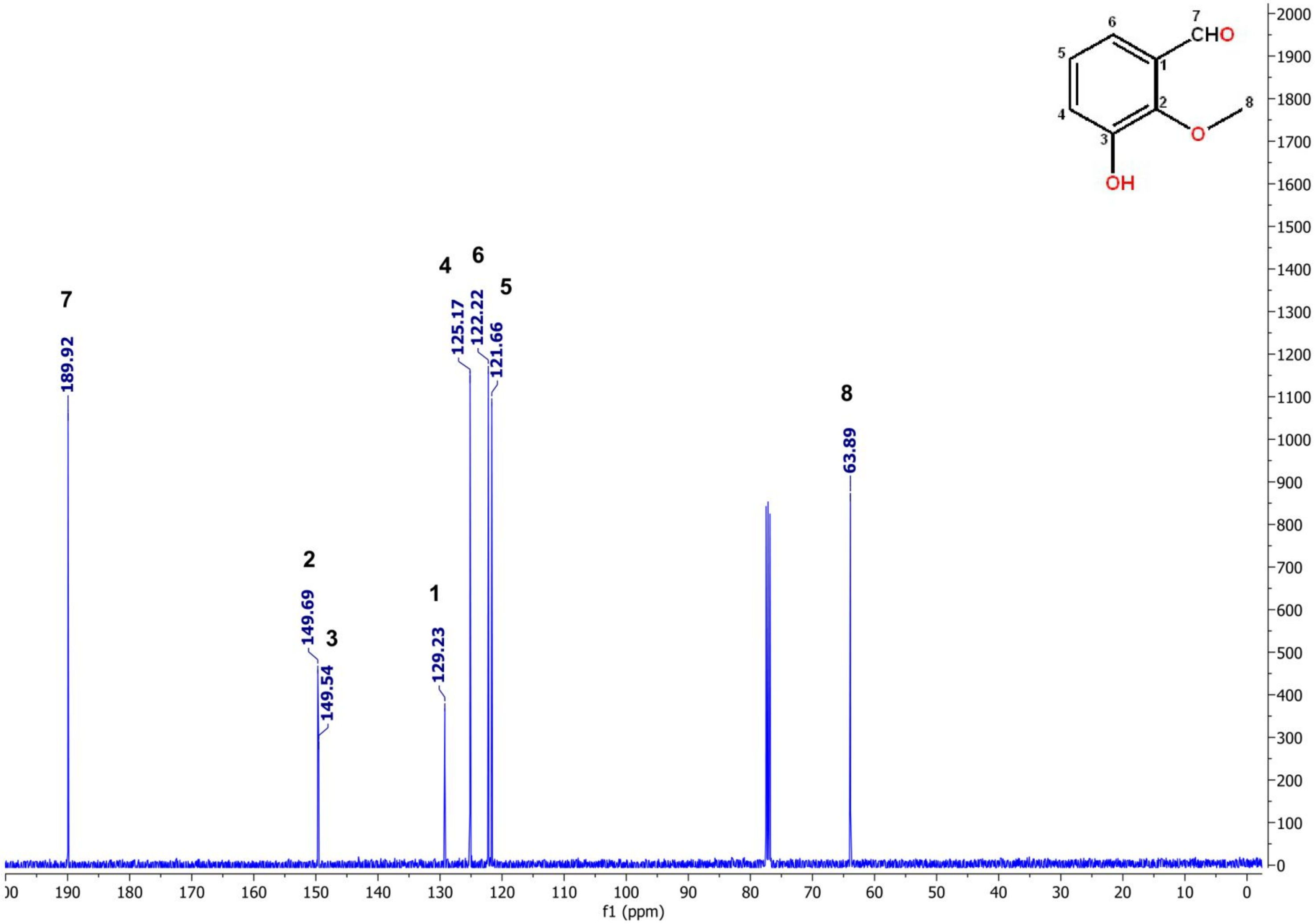

Figure S16. ^{13}C NMR spectrum of 3-hydroxy-2-methoxybenzaldehyde (**11**)

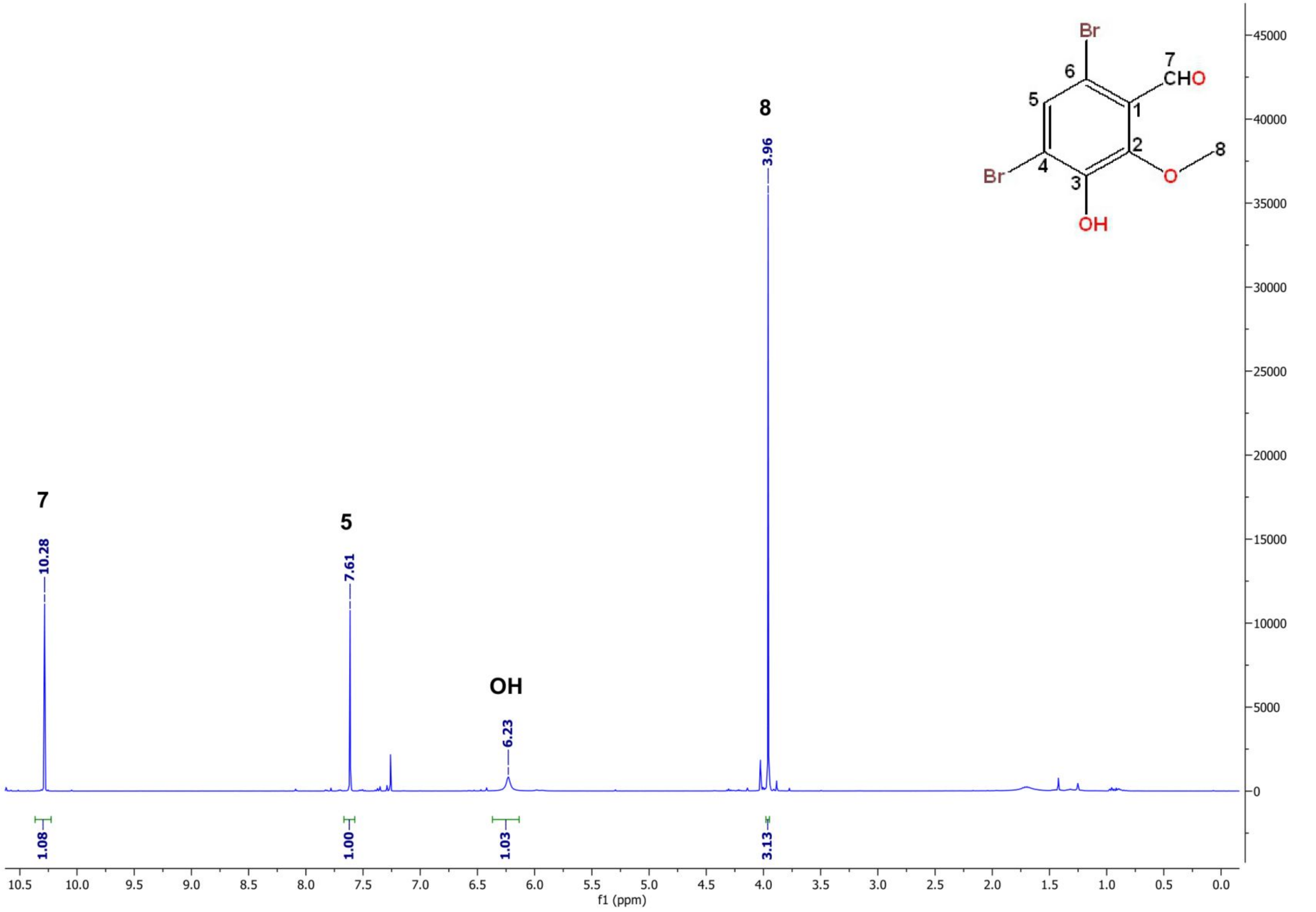

Figure S17. ^{1}H NMR spectrum of 4,6-dibromo-3-hydroxy-2-methoxybenzaldehyde (**12**)

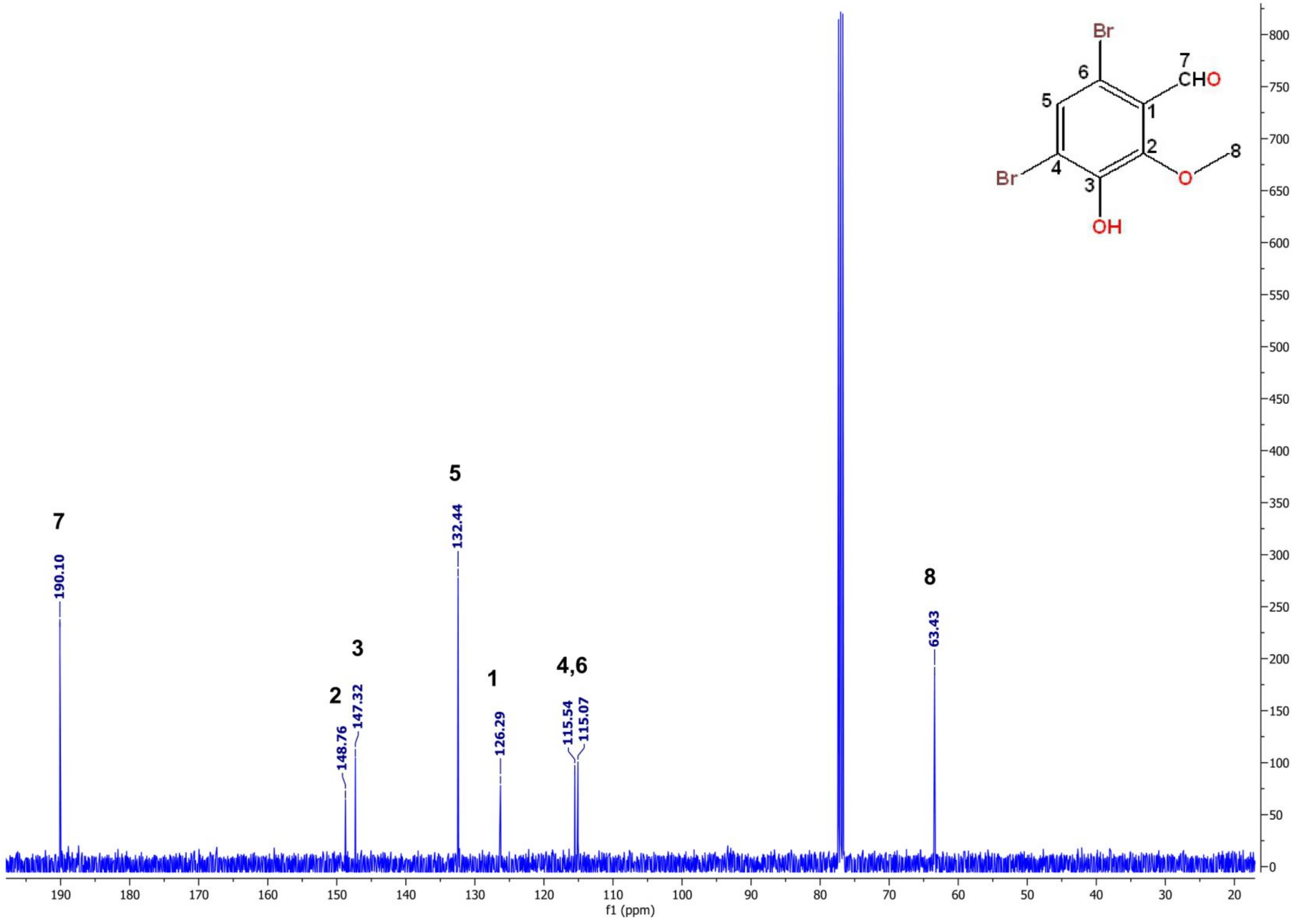

Figure S18. ^{13}C NMR spectrum of 4,6-dibromo-3-hydroxy-2-methoxybenzaldehyde (**12**)

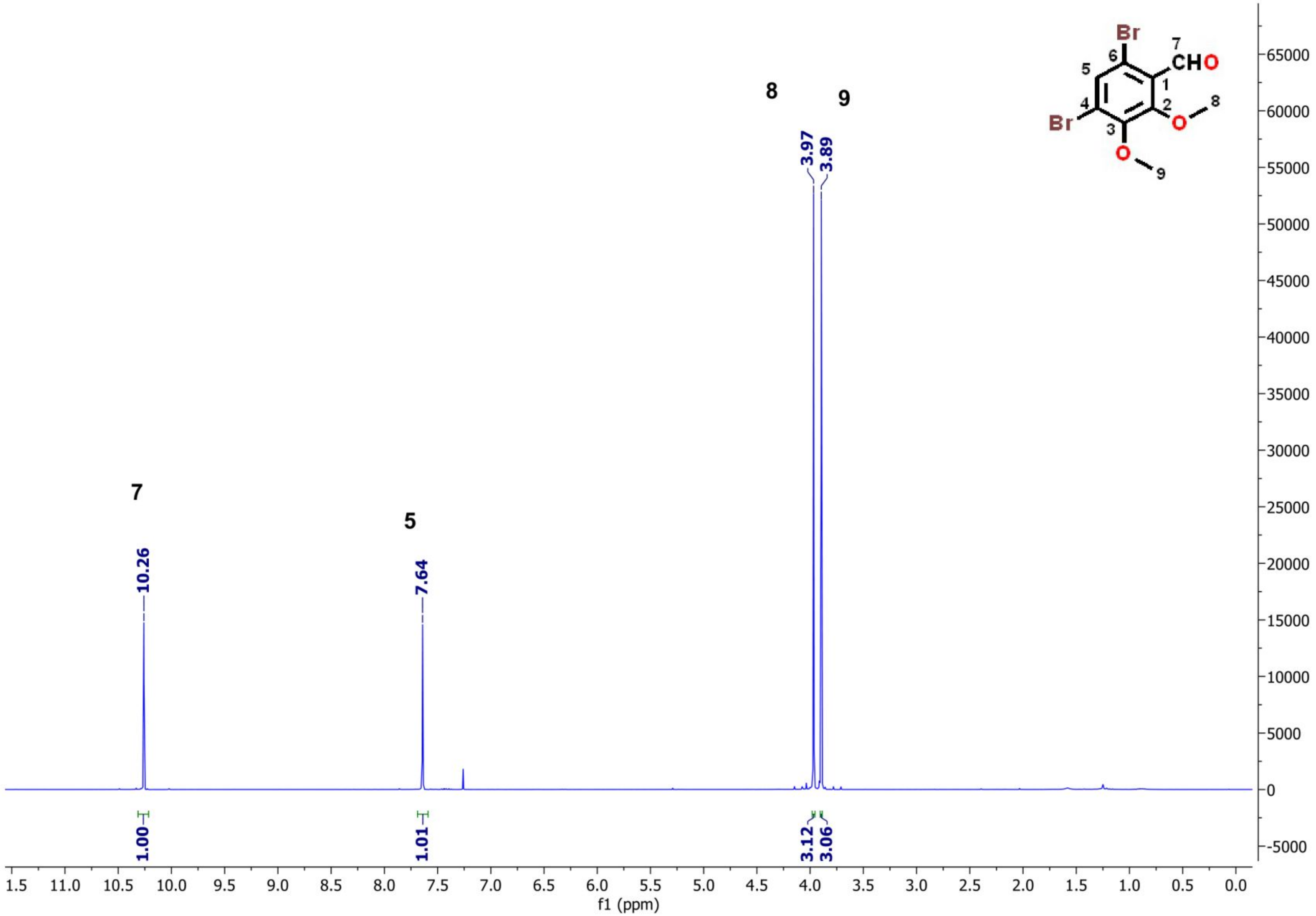

Figure S19. ^{1}H NMR spectrum of 4,6-dibromo-2,3-dimethoxybenzaldehyde (**13**)

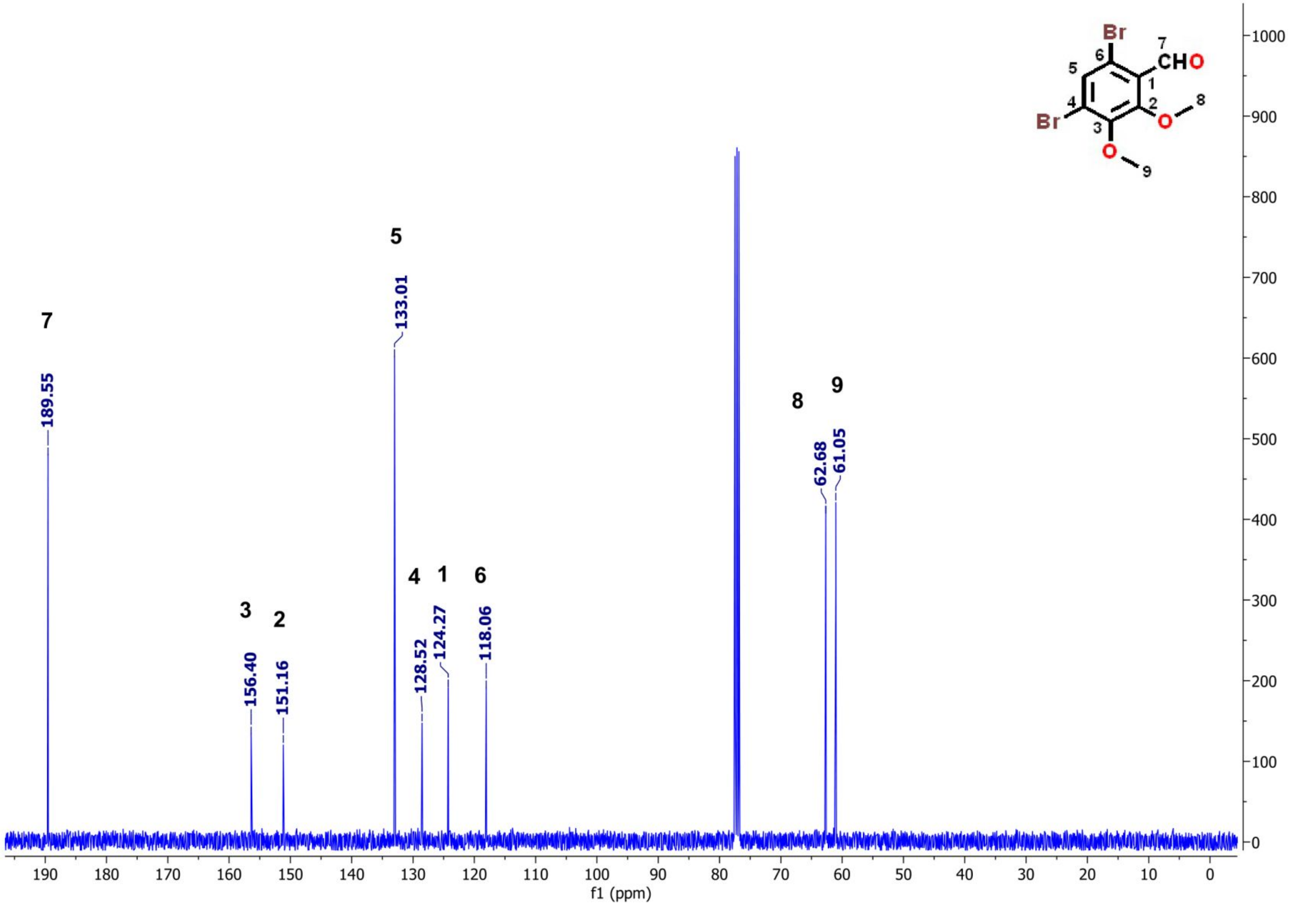

Figure S20. ^{13}C NMR spectrum of 4,6-dibromo-2,3-dimethoxybenzaldehyde (**13**)

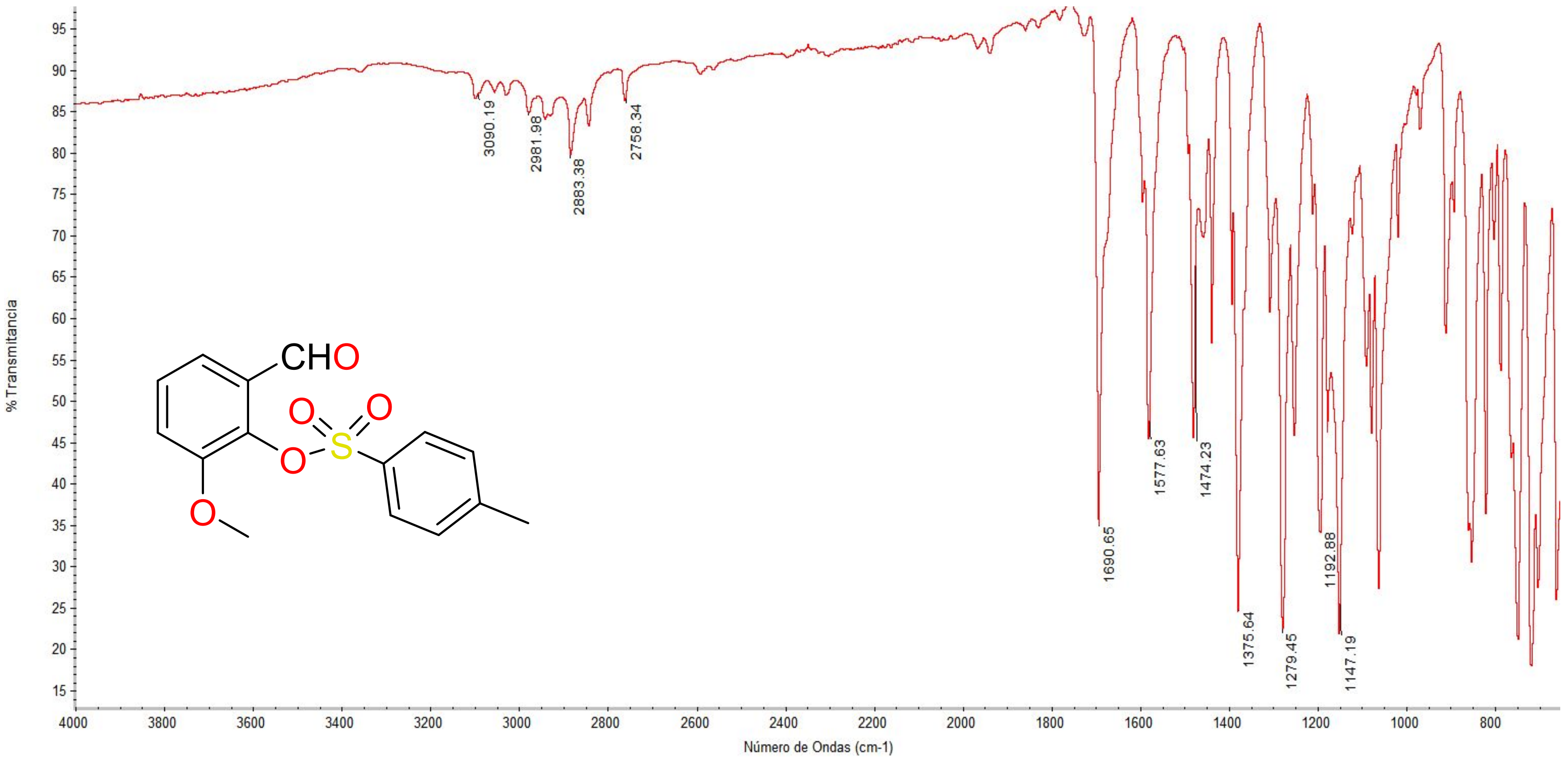

Figure S21. FTIR spectrum of 2-formyl-6-methoxyphenyl 4-methylbenzenesulfonate (**2**)

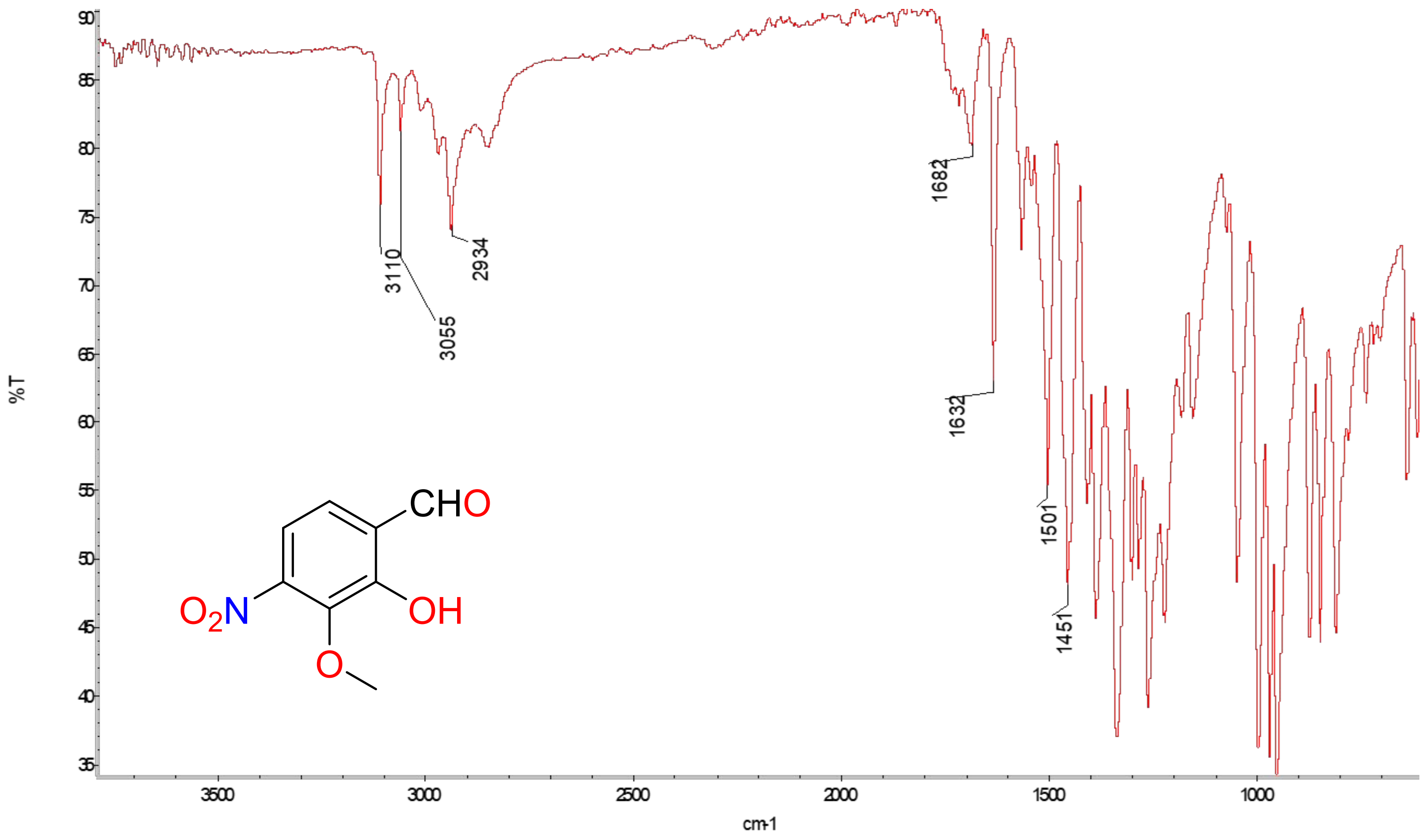

Figure S22. FTIR spectrum of 2-hydroxy-3-methoxy-4-nitrobenzaldehyde (**3**)

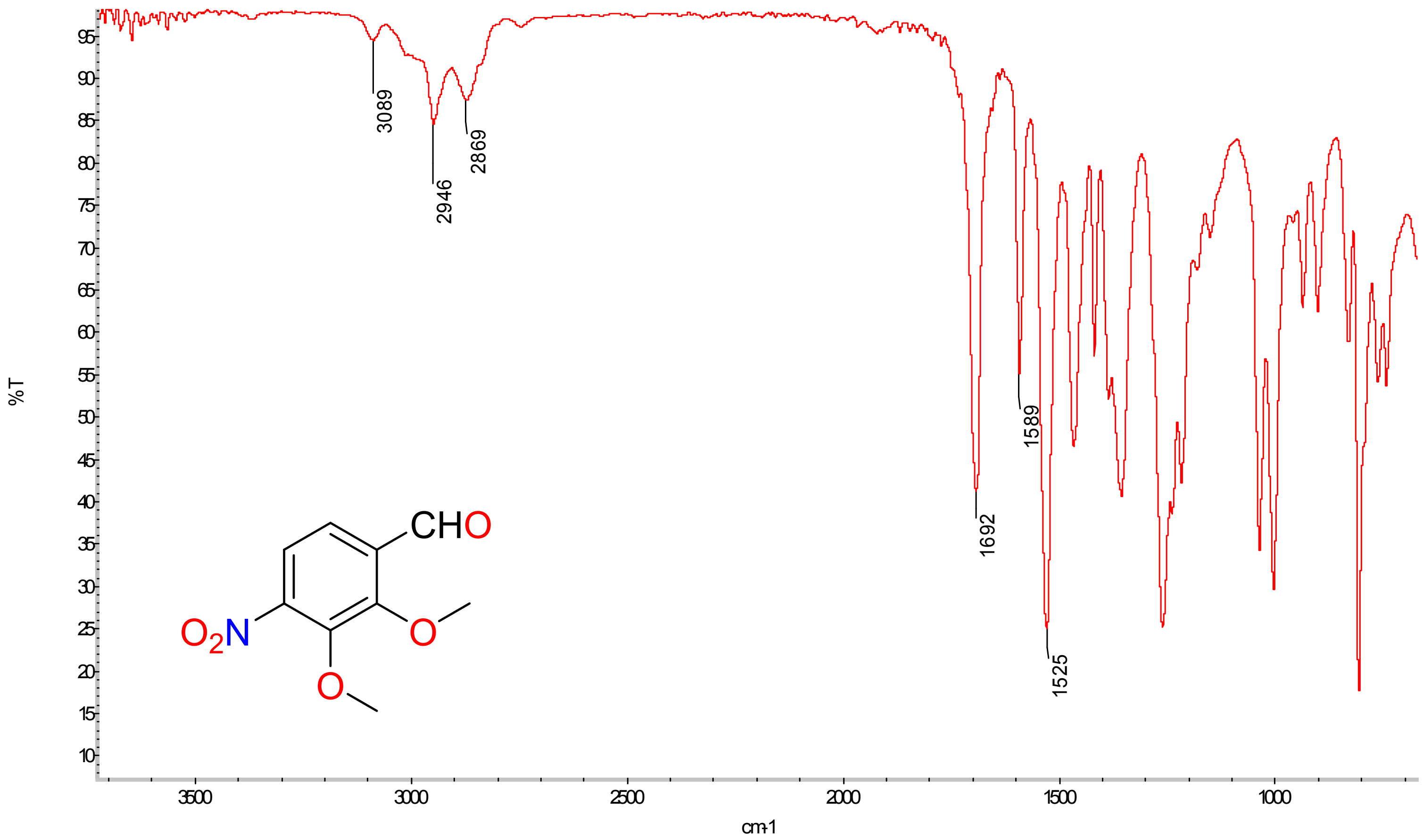

Figure S23. FTIR spectrum of 2,3-dimethoxy-4-nitrobenzaldehyde (**4**)

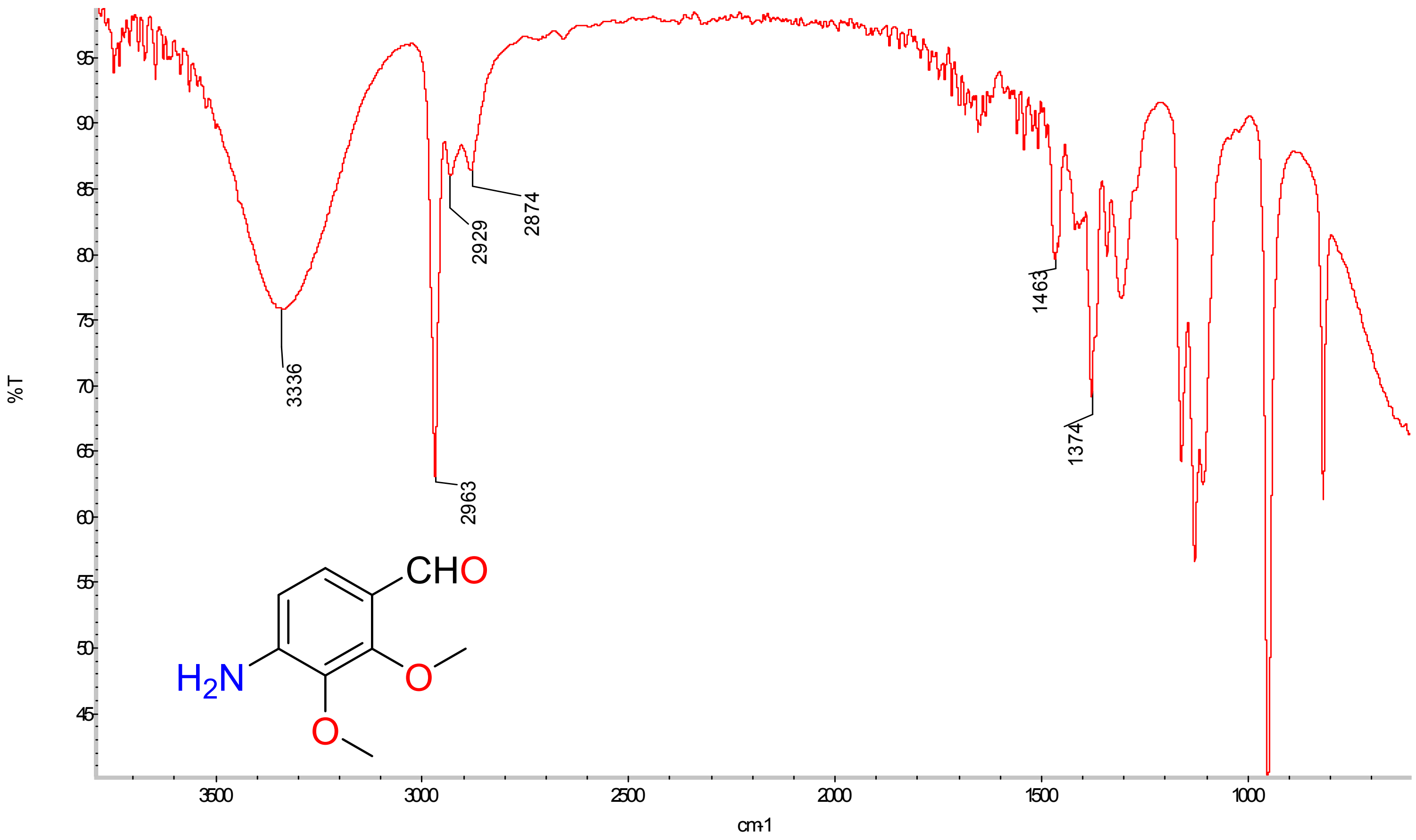

Figure S24. FTIR spectrum of 4-amino-2,3-dimethoxybenzaldehyde (**5**)

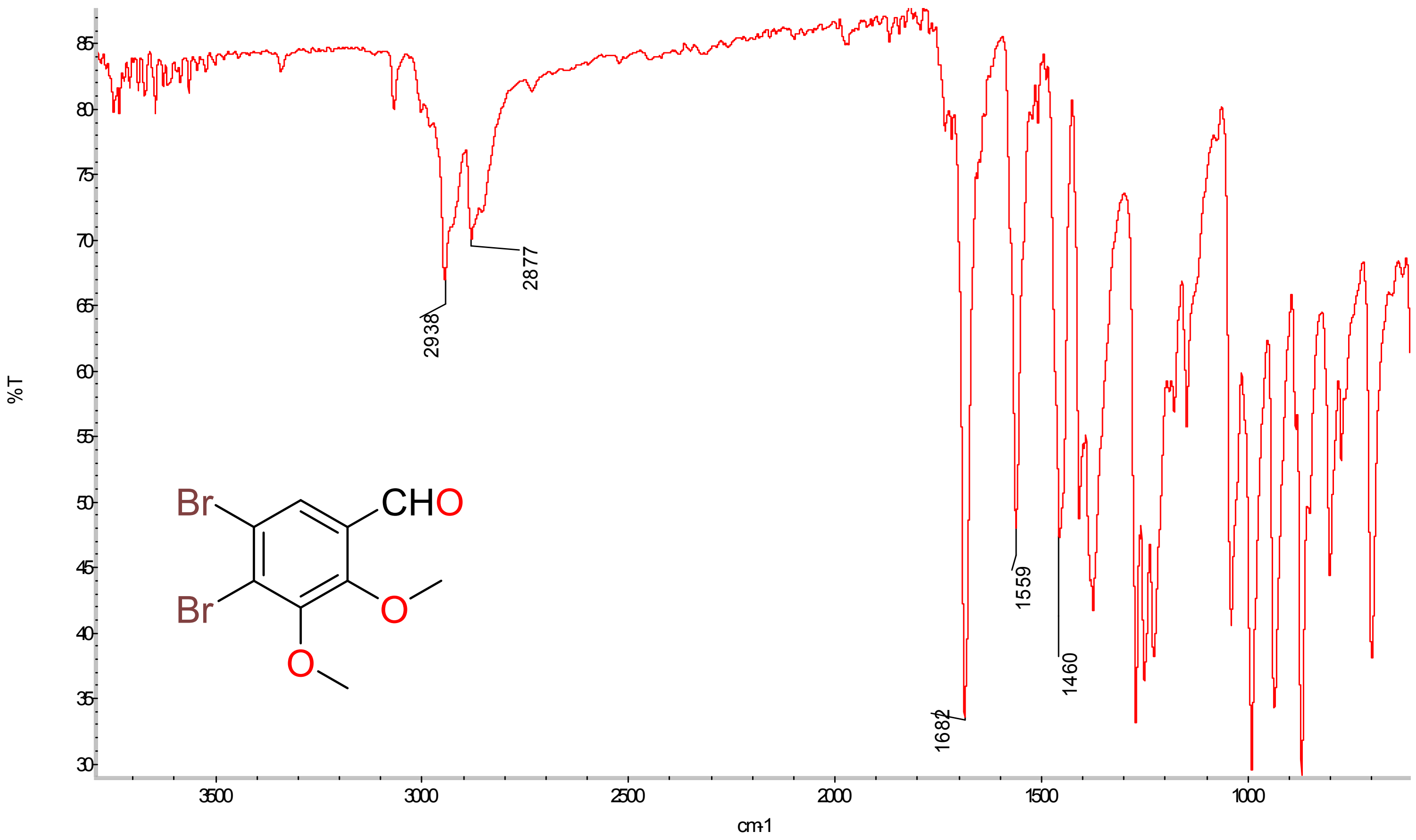

Figure S25. FTIR spectrum of 4,5-dibromo-2,3-dimethoxybenzaldehyde (**6**)

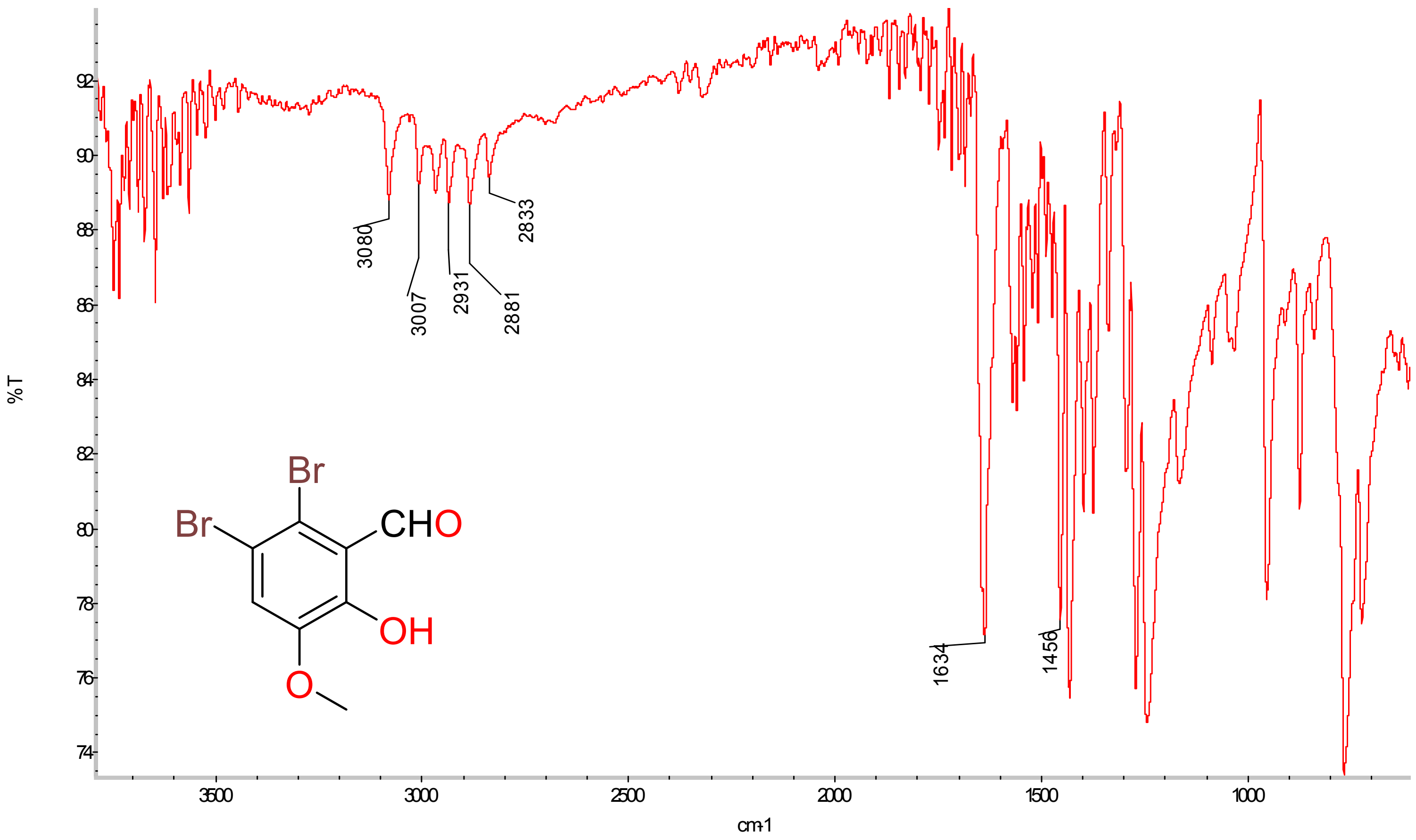

Figure S26. FTIR spectrum of 2,3-dibromo-6-hydroxy-5-methoxybenzaldehyde (**8**)

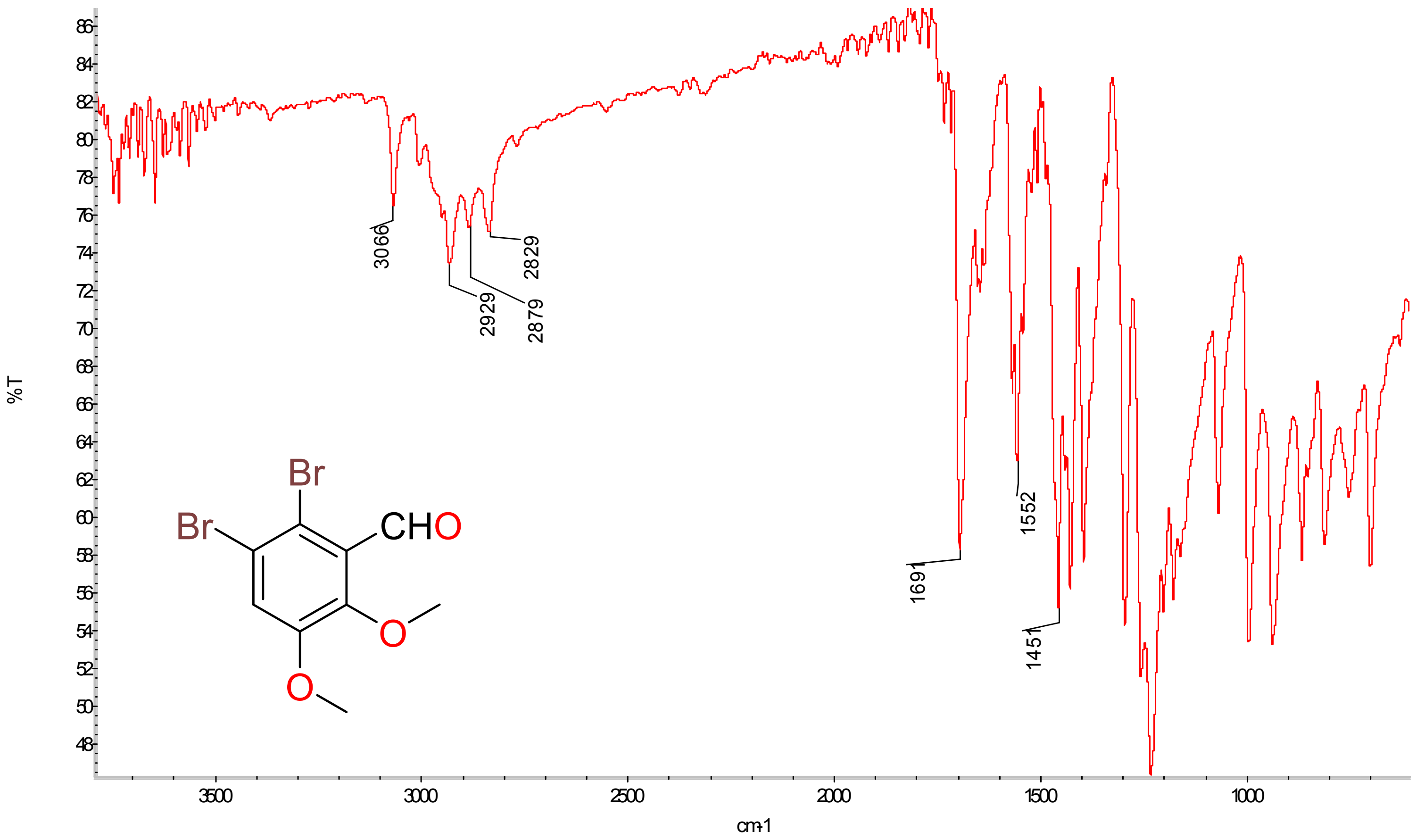

Figure S27. FTIR spectrum of 2,3-dibromo-5,6-dimethoxybenzaldehyde (**9**)

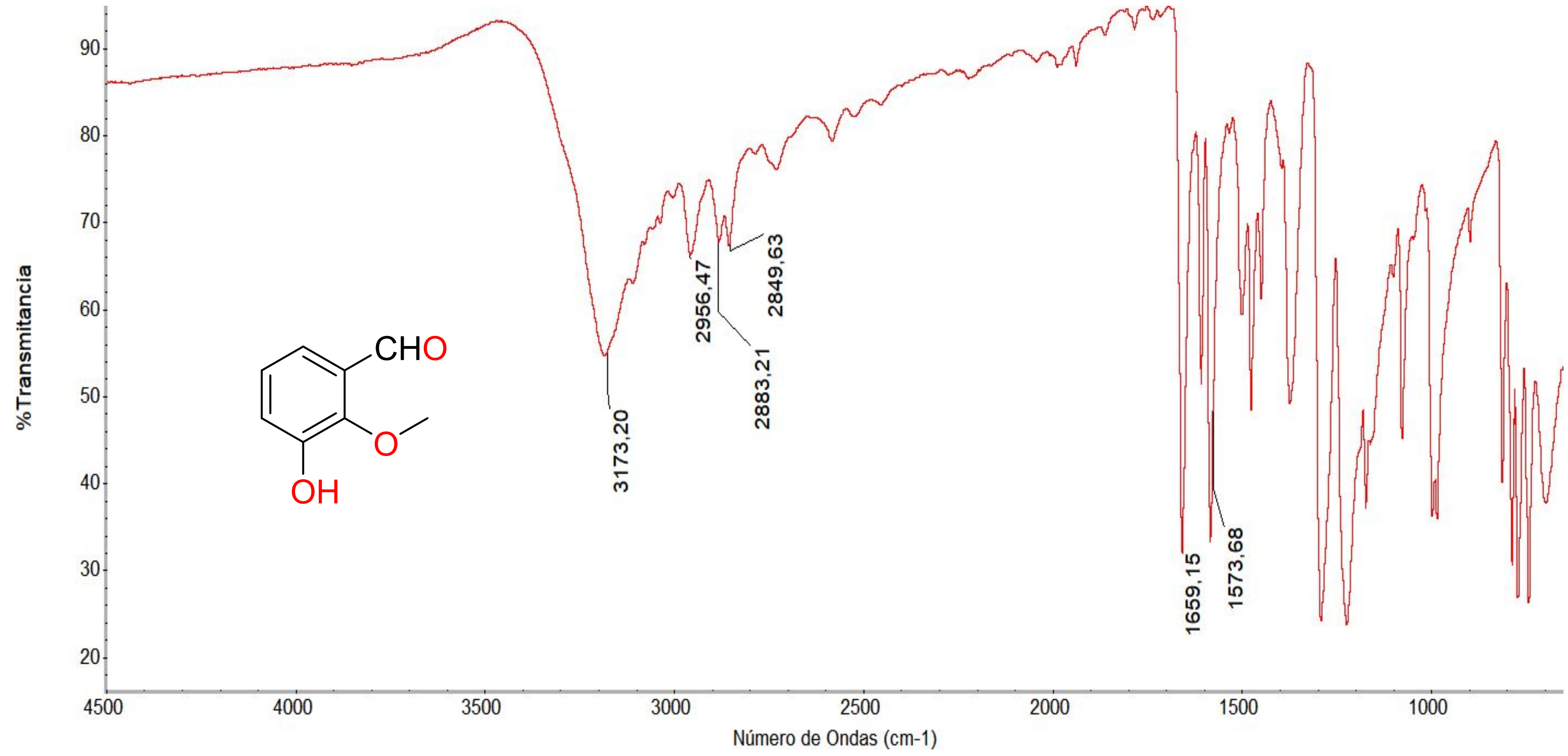

Figure S28. FTIR spectrum of 3-hydroxy-2-methoxybenzaldehyde (**11**)

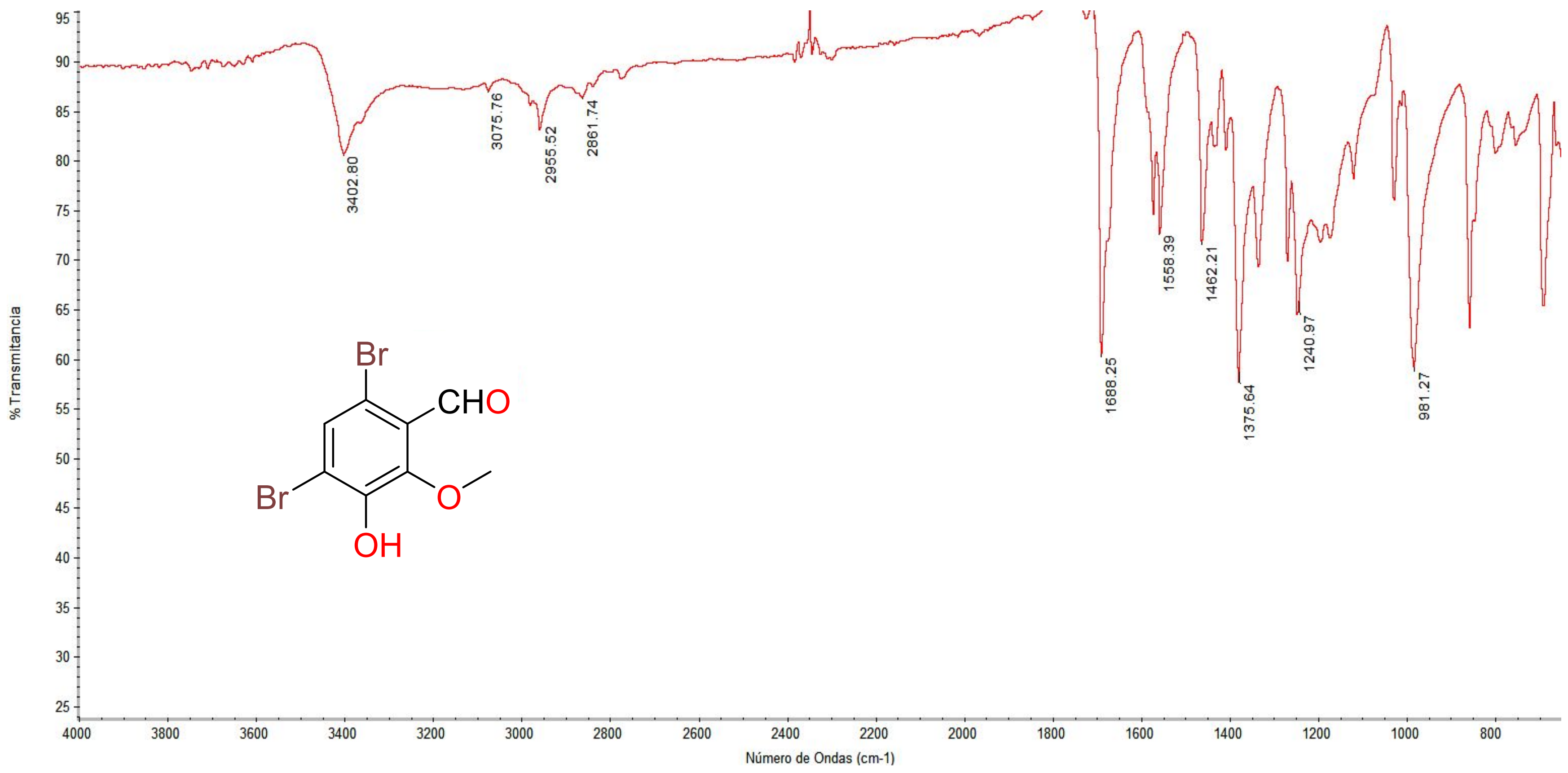

Figure S29. FTIR spectrum of 4,6-dibromo-3-hydroxy-2-methoxybenzaldehyde (**12**)

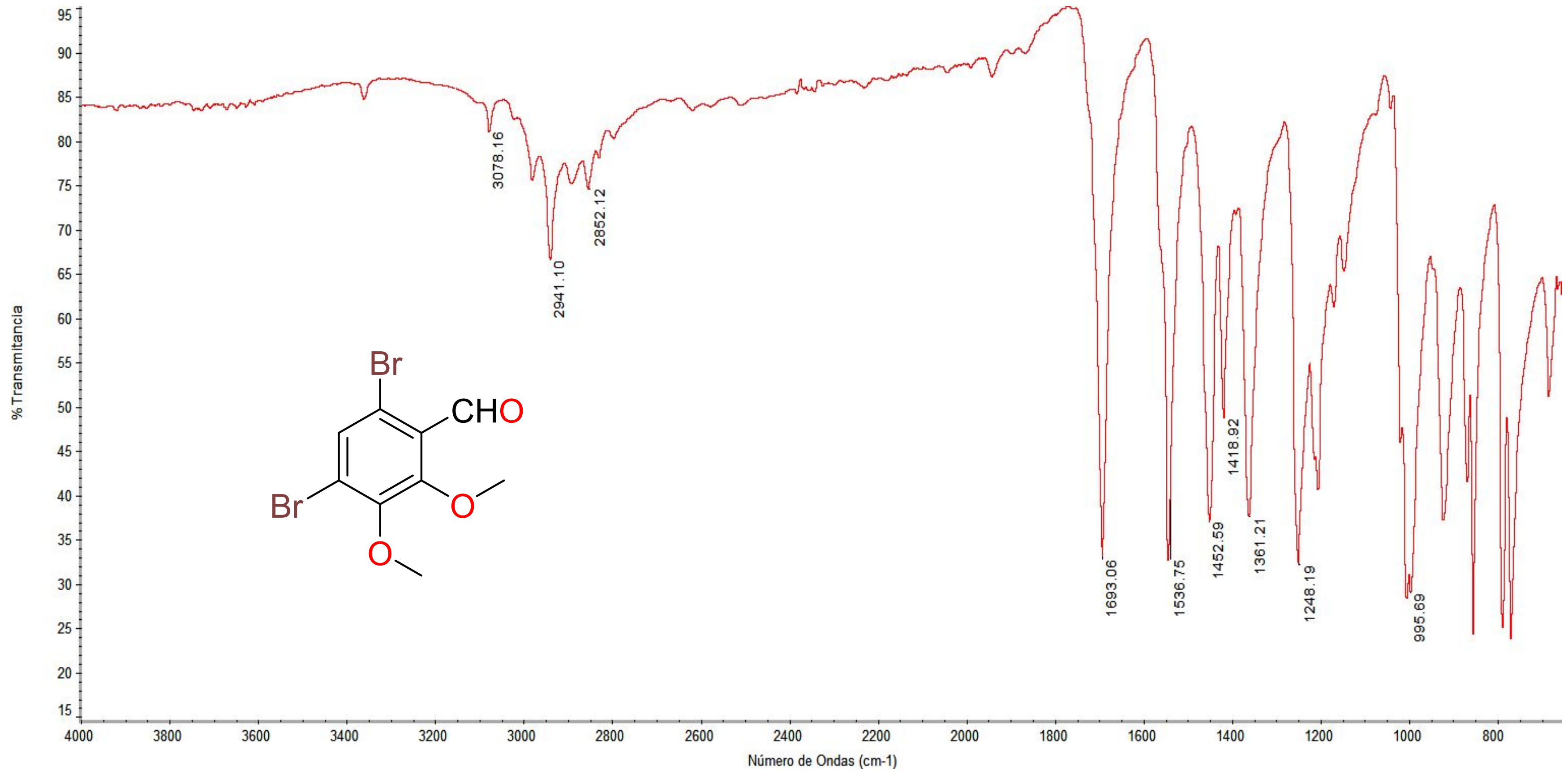

Figure S30. FTIR spectrum of 4,6-dibromo-2,3-dimethoxybenzaldehyde (**13**)

Table S1. Relevant experimental and theoretical bond length (Å), bond angles (°) and dihedral angles (°) for DMB's

	IB1	IB2	IB3		IB1	IB2	IB3
**Bond length**				**Dihedral angles**			
$Br_1 - C_4$	1.876	1.892	–	$C_8 - O_2 - C_2 - C_1$	-104.1	-105	104.4
$Br_2 - C_5$	1.885	–	–	$C_8 - O_2 - C_2 - C_3$	79.6	78.8	-78.5
$O_1 - C_7$	1.198	1.176	1.191	$C_9 - O_3 - C_3 - C_2$	83.8	82.8	176.4
$O_2 - C_2$	1.373	1.370	1.375	$C_9 - O_3 - C_3 - C_4$	-99.2	-99.2	-4.9
$O_2 - C_8$	1.436	1.431	1.446	$C_6 - C_1 - C_2 - O_2$	-177.7	-177	176.7
$O_3 - C_3$	1.374	1.358	1.368	$C_6 - C_1 - C_2 - C_3$	-1.4	-1.7	-0.4
$O_3 - C_9$	1.439	1.439	1.428	$C_7 - C_1 - C_2 - O_2$	3.5	3.4	-2
$C_1 - C_2$	1.402	1.405	1.401	$C_7 - C_1 - C_2 - C_3$	179.8	179.2	-179
$C_1 - C_6$	1.397	1.392	1.417	$C_2 - C_1 - C_6 - C_5$	0.6	0.9	-0.7
$C_1 - C_7$	1.528	1.490	1.486	$C_7 - C_1 - C_6 - C_5$	179.4	179.9	177.9
$C_2 - C_3$	1.386	1.392	1.392	$C_2 - C_1 - C_7 - O_1$	177.9	-173	-162
$C_3 - C_4$	1.397	1.385	1.377	$C_6 - C_1 - C_7 - O_1$	-0.8	7.9	19.3
$C_4 - C_5$	1.413	1.383	1.397	$O_2 - C_2 - C_3 - O_3$	-6	-5.1	1.9
$C_5 - C_6$	1.366	1.366	1.380	$O_2 - C_2 - C_3 - C_4$	177	176.8	-176
$Br_2 - C_6$	–	1.901	1.887	$C_1 - C_2 - C_3 - O_3$	177.8	179.1	179
$Br_1 - C_5$	–	–	1.898	$C_1 - C_2 - C_3 - C_4$	0.8	1	0.2
**Bond angles**				$O_3 - C_3 - C_4 - Br_1$	5.3	4.7	-
$C_2 - O_2 - C_8$	115.1	115.2	114.2	$O_3 - C_3 - C_4 - C_5$	-176.4	-177	-177
$C_3 - O_3 - C_9$	113.8	114.6	117.1	$C_2 - C_3 - C_4 - Br_1$	-177.7	-177	–
$C_2 - C_1 - C_6$	119.1	116.9	118.7	$C_2 - C_3 - C_4 - C_5$	0.7	0.5	1.1
$C_2 - C_1 - C_7$	120.6	117.4	116.7	$Br_1 - C_4 - C_5 - Br_2$	-3.2	–	–
$C_6 - C_1 - C_7$	120.4	125.7	124.6	$Br_1 - C_4 - C_5 - C_6$	176.8	176.6	–
$O_2 - C_2 - C_1$	119.3	–	119.1	$C_3 - C_4 - C_5 - Br_2$	178.4	–	–
$O_2 - C_2 - C_3$	120.1	–	119.6	$C_3 - C_4 - C_5 - C_6$	-1.5	-1.2	-2.3
$C_1 - C_2 - C_3$	120.5	122.3	121.2	$Br_2 - C_5 - C_6 - C_1$	-179.1	–	–
$O_3 - C_3 - C_2$	120.8	–	115.7	$C_4 - C_5 - C_6 - C_1$	0.9	0.5	2
$O_3 - C_3 - C_4$	119.4	–	124.6	$C_2 - C_1 - C_6 - Br_2$	–	-179	-178
$C_2 - C_3 - C_4$	119.7	117.5	119.7	$C_7 - C_1 - C_6 - Br_2$	–	-0.7	0.5
$Br_1 - C_4 - C_3$	119.3	119.1	–	$C_4 - C_5 - C_6 - Br_2$	–	-178	179.4
$Br_1 - C_4 - C_5$	121.0	118.9	–	$C_3 - C_4 - C_5 - Br_1$	–	–	177.7

$C_3 - C_4 - C_5$	119.7	122.0	119.7
$Br_2 - C_5 - C_4$	121.3	–	–
$Br_2 - C_5 - C_6$	118.9	–	–
$C_4 - C_5 - C_6$	119.8	119.1	121.7
$C_1 - C_6 - C_5$	121.1	122.3	119.0
$O_1 - C_7 - C_1$	119.0	–	127.3
$Br_1 - C_5 - C_4$	–	–	116.6
$Br_1 - C_5 - C_6$	–	–	121.7
$Br_2 - C_6 - C_1$	–	122.0	121.4
$Br_2 - C_6 - C_5$	–	115.8	119.6

$Br_1 - C_5 - C_6 - Br_2$	–	–	-0.5
$Br_1 - C_5 - C_6 - C_1$	–	–	-177

Table S2. QTAIM topological parameters to the intermolecular interactions observed in IB1, IB2, and IB3 compounds.

Interaction	D–H···A (Å)	D–H–A (°)	$\rho(r)$ (a.u.)	$\nabla^2\rho$ (a.u.)	$G(r)$ (a.u.)	$v(r)$ (a.u.)	$h(r)$ (a.u.)	$\frac{\lvert v\rvert}{G}$	BE (kcal/mol)	Interaction Type
					**IB1**					
C_7–H···O_1 (A)	2.645	125.859	0.0068	0.0251	0.0053	-0.0043	0.0010	0.8	-0.765	van der Waals
C_8–H···O_1 (B)	2.575	172.754	0.0063	0.0221	0.0046	-0.0038	0.0009	0.8	-0.656	van der Waals
C_7–O_1···C_7···O_1 (C)	-	-	0.0038	0.0139	0.0025	-0.0016	0.0010	0.6	-0.098	van der Waals
C_9–H···O_3 (D)	2.702	161.944	0.0057	0.0185	0.0040	-0.0034	0.0006	0.8	-0.522	van der Waals
C_8–H···O_2 (E)	2.817	143.290	0.0046	0.0159	0.0034	-0.0028	0.0006	0.8	-0.286	van der Waals
O_2···O_3 (F)	3.411	-	0.0031	0.0138	0.0028	-0.0022	0.0006	0.8	0.060	van der Waals
C_4–Br_1···C_9 (G)	3.905	102.760	0.0038	0.0123	0.0024	-0.0016	0.0007	0.7	-0.096	van der Waals
Br_1···C_4 (H)	3.672	-	0.0061	0.0171	0.0035	-0.0028	0.0008	0.8	-0.619	van der Waals
C_4···C_1 (I)	3.518	-	0.0053	0.0138	0.0030	-0.0025	0.0005	0.8	-0.429	van der Waals
C_7–H···H–C_8 (J)	2.775	-	0.0023	0.0079	0.0016	-0.0011	0.0004	0.7	0.220	van der Waals
C_6···O_1 (K)	3.542	-	0.0038	0.0113	0.0023	-0.0018	0.0005	0.8	-0.102	van der Waals
Br_2···C_6 (L)	3.842	-	0.0035	0.0110	0.0022	-0.0016	0.0006	0.7	-0.047	van der Waals
C_7–O_1···Br_2	3.286	-	0.0061	0.0248	0.0051	-0.0040	0.0011	0.8	-0.628	van der Waals
Br_1···Br_1	3.644	-	0.0062	0.0218	0.0043	-0.0031	0.0012	0.7	-0.634	van der Waals
					**IB2**					
C_7–H···O_1 (A)	2.706	144.724	0.0057	0.0187	0.0041	-0.0035	0.0006	0.9	-0.523	van der Waals
C_8–H···O_1 (B)	2.646	145.187	0.0060	0.0198	0.0043	-0.0036	0.0007	0.8	-0.596	van der Waals
C_8–H···O_2 (C)	3.151	117.978	0.0026	0.0108	0.0021	-0.0015	0.0006	0.7	0.166	van der Waals
C_8–H···O_3 (D)	2.681	168.029	0.0055	0.0185	0.0040	-0.0034	0.0006	0.8	-0.495	van der Waals
C_8–H···H–C_8 (E)	2.540	-	0.0034	0.0116	0.0024	-0.0018	0.0005	0.8	-0.027	van der Waals
C_8–H···H–C_9 (F)	2.590	-	0.0032	0.0114	0.0023	-0.0017	0.0006	0.7	0.029	van der Waals
C_9–H···Br_2 (G)	3.444	118.324	0.0033	0.0104	0.0020	-0.0014	0.0006	0.7	0.008	van der Waals
					**IB3**					
C_9–H···O_1 (A)	2.496	160.445	0.0075	0.0268	0.0056	-0.0045	0.0011	0.8	-0.936	van der Waals
C_4–H···O_1 (B)	3.245	105.825	0.0027	0.0103	0.0020	-0.0014	0.0006	0.7	0.135	van der Waals
C_8–H···Br_1 (C)	3.220	160.391	0.0043	0.0120	0.0024	-0.0019	0.0006	0.8	-0.211	van der Waals
C_8–H···O_2 (D)	2.837	110.449	0.0056	0.0219	0.0045	-0.0035	0.0010	0.8	-0.501	van der Waals
C_9–H···O_3 (E)	2.670	155.949	0.0064	0.0200	0.0045	-0.0039	0.0006	0.9	-0.685	van der Waals
Br_2···Br_2 (F)	3.958	-	0.0053	0.0152	0.0030	-0.0021	0.0008	0.7	-0.440	van der Waals
Br_1···C_5 (G)	-	-	0.0069	0.0201	0.0041	-0.0032	0.0009	0.8	-0.791	van der Waals
C_5...C_1 (H)	-	-	0.0055	0.0146	0.0031	-0.0026	0.0005	0.8	-0.485	van der Waals
C_4...C_2–C_3 (I)	-	-	0.0055	0.0141	0.0030	-0.0025	0.0005	0.8	-0.488	van der Waals
O_2···C_2–C_3 (J)	-	-	0.0045	0.0147	0.0031	-0.0025	0.0006	0.8	-0.257	van der Waals
C_8–H···Br_1 (K)	3.369	156.070	0.0032	0.0099	0.0019	-0.0013	0.0006	0.7	0.039	van der Waals
C_8–H···Br_2 (L)	3.231	105.540	0.0054	0.0194	0.0037	-0.0026	0.0011	0.7	-0.460	van der Waals
O_3···Br_2 (M)	3.177	-	0.0090	0.0346	0.0075	-0.0063	0.0012	0.8	-1.258	van der Waals
